# Pan-Asian adapted ESMO Clinical Practice Guidelines for the diagnosis, treatment and follow-up of patients with endometrial cancer

**DOI:** 10.1016/j.esmoop.2022.100774

**Published:** 2023-01-23

**Authors:** S. Koppikar, A. Oaknin, K. Govind Babu, D. Lorusso, S. Gupta, L.-Y. Wu, W. Rajabto, K. Harano, S.-H. Hong, R.A. Malik, H. Strebel, I.M. Aggarwal, C.-H. Lai, T. Dejthevaporn, S. Tangjitgamol, W.F. Cheng, W.Y. Chay, D. Benavides, N.M. Hashim, Y.W. Moon, M. Yunokawa, T.D. Anggraeni, W. Wei, G. Curigliano, A. Maheshwari, U. Mahantshetty, S. Sheshadri, S. Peters, T. Yoshino, G. Pentheroudakis

**Affiliations:** 1 Department of Medical Oncology, Lilavati Hospital and Research Centre, Mumbai, India; 2 Department of Medical Oncology, Bombay Hospital Institute of Medical Sciences, Mumbai, India; 3 Gynaecologic Cancer Programme, Vall d’Hebron Institute of Oncology (VHIO), Hospital Universitari Vall d’Hebron, Vall d’Hebron Barcelona Hospital Campus, Barcelona, Spain; 4 Department of Medical Oncology, HCG Hospital and St. Johns Medical College, Bengaluru, India; 5 Department of Life Science and Public Health, Catholic University of Sacred Heart, Largo Agostino Gemelli, Rome; 6 Department of Women and Child Health, Division of Gynaecologic Oncology, Fondazione Policlinico Universitario Agostino Gemelli IRCCS, Rome, Italy; 7 Department of Medical Oncology, Tata Memorial Centre and Homi Bhabha National Institute, Mumbai, India; 8 Department of Gynecologic Oncology, National Cancer Center/National Clinical Research Center for Cancer/Cancer Hospital, Chinese Academy of Medical Sciences and Peking Union Medical College, Beijing, China; 9 Division of Gynecologic Oncology, Department of Obstetrics and Gynecology, Dr. Cipto Mangunkusumo General Hospital/Faculty of Medicine Universitas Indonesia, Jakarta, Indonesia; 10 Department of Medical Oncology, National Cancer Center Hospital East, Kashiwa, Japan; 11 Department of Internal Medicine, Seoul St. Mary’s Hospital, College of Medicine, The Catholic University of Korea, Seoul, Republic of Korea; 12 Clinical Oncology Unit, Faculty of Medicine, University of Malaya, Kuala Lumpur, Malaysia; 13 Division of Medical Oncology, Department of Internal Medicine, University of the Philippines, Philippine General Hospital, Manila, The Philippines; 14 Department of Gynaecologic Oncology, KK Women’s and Children’s Hospital, Singapore, Singapore; 15 Department of Obstetrics and Gynecology, Chang Gung Memorial Hospital, Linkou Branch, Taoyuan, Taiwan; 16 Medical Oncology Unit, Faculty of Medicine, Ramathibodi Hospital, Mahidol University, Bangkok, Thailand; 17 Department of Obstetrics and Gynecology, Faculty of Medicine Vajira Hospital, Navamindradhiraj University, Bangkok, Thailand; 18 Obstetrics and Gynecology Center, Medpark Hospital, Bangkok, Thailand; 19 Department of Obstetrics and Gynecology, National Taiwan University Hospital, Taipei, Taiwan; 20 Division of Medical Oncology, National Cancer Centre, Singapore, Singapore; 21 Division of Gynecologic Oncology, Department of Obstetrics and Gynecology, U.P. College of Medicine and Philippine General Hospital, Manila, The Philippines; 22 Oncology and Radiotherapy Department, KPJ Johor Specialist Hospital, Johor Bahru, Malaysia; 23 Department of Hematology and Oncology, CHA Bundang Medical Center (CBMC), CHA University, Seongnam, Gyeonggi-do, Republic of Korea; 24 Department of Gynecology and Medical Oncology, The Cancer Institute Hospital of Japanese Foundation for Cancer Research (JFCR), Tokyo, Japan; 25 Department of Gynecologic Oncology, State Key Laboratory of Oncology in South China, Collaborative Innovation Centre for Cancer Medicine, Sun Yat-Sen University Cancer Center, Guangzhou, Guangdong, China; 26 European Institute of Oncology, IRCCS, Milano, Italy; 27 Department of Oncology and Hemato-Oncology, University of Milano, Milano, Italy; 28 Department of Gynecologic Oncology, Tata Memorial Centre and Homi Bhabha National Institute, Mumbai, India; 29 Department of Radiation Oncology, Homi Bhabha Cancer Hospital and Research Hospital, Vishakhapatnam, India; 30 Department of Pathology, Kidwai Memorial Institute of Oncology, Bengaluru, India; 31 Oncology Department, Lausanne University Hospital (CHUV), Lausanne, Switzerland; 32 Department of Gastroenterology and Gastrointestinal Oncology, National Cancer Center Hospital East, Kashiwa, Japan; 33 ESMO, Lugano, Switzerland

**Keywords:** ESMO, guidelines, Pan-Asian, endometrial cancer, treatment

## Abstract

The most recent version of the European Society for Medical Oncology (ESMO) Clinical Practice Guidelines for the diagnosis, treatment and follow-up of patients with endometrial cancer was published in 2022. It was therefore decided, by both the ESMO and the Indian Society of Medical and Paediatric Oncology (ISMPO), to convene a virtual meeting in July 2022 to adapt the ESMO 2022 guidelines to take into account the variations in the management of endometrial cancer in Asia. These guidelines represent the consensus opinion of a panel of Asian experts representing the oncological societies of China (CSCO), India (ISMPO), Indonesia (ISHMO), Japan (JSMO), Korea (KSMO), Malaysia (MOS), the Philippines (PSMO), Singapore (SSO), Taiwan (TOS) and Thailand (TSCO). Voting was based on scientific evidence and was conducted independently of the current treatment practices and treatment access constraints in the different Asian countries, which were discussed when appropriate. The aim of this guideline manuscript is to provide guidance for the optimisation and harmonisation of the management of patients with endometrial cancer across the different regions of Asia, drawing on the evidence provided by Western and Asian trials whilst respecting the variations in clinical presentation, diagnostic practices including molecular profiling and disparities in access to therapeutic options, including drug approvals and reimbursement strategies.

## Highlights

• This article provides the ESMO recommendations for the treatment of endometrial cancer adapted for patients in Asian countries.
• The lack of availability of certain tests and treatments in some Asian countries is discussed for few recommendations.
• The aim is to encourage evidence-based practices in the management of endometrial cancer in the different regions of Asia.

## Introduction

Cancer of the corpus uteri (endometrial cancer) is the most common gynaecological malignancy in high- and intermediate-income countries.^1^^,^^2^ In 2020, endometrial cancer was the sixth most commonly diagnosed cancer in women, with 417 367 new cases recorded, accounting for 2.2% of the new cancers diagnosed worldwide. Approximately 40% of these new cases occurred in Asia, with China, where endometrial cancer is the third most common female malignancy, accounting for nearly half (81 964) of the cases.^3^ Endometrial cancer was in turn responsible for 97 370 cancer deaths representing 1% of all cancer deaths worldwide.^4^

Although endometrial cancer has a higher incidence in Western countries than in Asia, the incidence is increasing worldwide. Risk factors that are associated with sporadic endometrial cancer include obesity (high body mass index), diabetes, polycystic ovary syndrome, early age at menarche, late menopause, infertility, menopausal estrogen therapy and the use of tamoxifen,^5^^,^^6^ whilst inherited endometrial cancer is linked to Lynch and Cowden syndromes.^7^

A rising trend in endometrial cancer is being observed in several Asian countries. The number of new cases of endometrial cancer in 2020 was 16 413 cases in India, 4524 cases in Thailand, 4374 cases in the Philippines, 3425 cases in South Korea, 1401 cases in Malaysia and 775 cases in Singapore.^8^ The increasing incidence is attributed to evolving lifestyle, younger age at menarche, late age at menopause and fewer children, especially in women living in urban areas.^9^^,^^10^

Although endometrial cancer occurs most frequently in postmenopausal women, there is a higher proportion of younger women being diagnosed with endometrial cancer in China,^11^^,^^12^ with ∼40% of patients diagnosed before their menopause compared with <25% of Western women.^13^ In Hong Kong, 65% of 1165 new cases of endometrial cancer diagnosed in 2018 occurred in women aged between 45 and 64 years (www3.ha.org.hk/cancereg).

The majority of endometrial cancers are diagnosed at an early stage and the 5-year overall survival rate for patients with localised disease is high (95%), However, endometrial cancers with high-risk factors such as high-grade serous pathology and TP53 mutation have a tendency to recur.^1^^,^^14^ Patients with recurrent endometrial cancer have a poor prognosis, with a 5-year overall survival of <20%, particularly in patients with metastatic disease.^15^

Guidelines and recommendations for the treatment and management of patients with endometrial cancer in Asia have been published for the Asia-Pacific region, India [National Cancer Grid (NCG) guidelines for endometrial cancer (tmc.gov.in)], Japan,^16^ Korea,^17^ Singapore,^18^ Taiwan,^19^ China, Thailand, the Philippines and Indonesia, and are important for the standardisation of diagnostic and treatment approaches. These guidelines aim to optimise clinical outcomes for what is a growing health care problem in each Asian country. The European Society for Medical Oncology (ESMO) guidelines for the diagnosis, treatment and follow-up of patients with endometrial cancer were published in 2022,^20^ and a decision was taken by ESMO and the Indian Society of Medical and Paediatric Oncology (ISMPO) that these guidelines should be adapted for the management and treatment of patients in Asian countries.

Consequently, representatives of ISMPO, ESMO, the Chinese Society of Clinical Oncology (CSCO), the Indonesian Society of Hematology and Medical Oncology (ISHMO), the Japanese Society of Medical Oncology (JSMO), the Korean Society of Medical Oncology (KSMO), the Malaysian Oncological Society (MOS), the Philippine Society of Medical Oncology (PSMO), the Singapore Society of Oncology (SSO), the Taiwan Oncology Society (TOS) and the Thai Society of Clinical Oncology (TSCO) convened for a virtual, ‘face-to-face’ working meeting on 9 July 2022, hosted by ISMPO, to adapt the recent ESMO Clinical Practice Guidelines^20^ for use in the clinical management of Asian patients with endometrial cancer. This manuscript summarises the Pan-Asian adapted guidelines developed at the meeting accompanied by the level of evidence (LoE), grade of recommendation (GoR) and percentage consensus reached for each recommendation.

## Methodology

This Pan-Asian adaptation of the current ESMO Clinical Practice Guidelines^20^ was prepared in accordance with the principles of ESMO standard operating procedures (http://www.esmo.org/Guidelines/ESMO-Guidelines-Methodology) and was an ISMPO–ESMO initiative endorsed by CSCO, ISHMO, JSMO, KSMO MOS, PSMO, SSO, TOS and TSCO.

An international panel of experts was selected from the ISPMO (*n* = 6), the ESMO (*n* = 6) and two experts representing each of the oncological societies of China (CSCO), Indonesia (ISHMO), Japan (JSMO), Korea (KSMO), Malaysia (MOS), the Philippines (PSMO), Singapore (SSO), Taiwan (TOS) and Thailand (TSCO). One expert from Thailand (ST) was member of the Thai Gynecologic Cancer Society endorsed by TSCO. Only two of the six expert members from the ISMPO (SG and KGB) were allowed to vote on the recommendations together with the experts from each of the nine other Asian oncology societies (*n* = 20). Among the six experts from ISMPO, three were medical oncologists and one a gynaecological oncologist, one a radiation oncologist and one a pathologist. The majority of experts from the other Asian societies were medical oncologists or gynaecological oncologists. None of the additional ISMPO members present and none of the ESMO experts were allowed to vote and were present only in an advisory role.

A modified Delphi process was used to review, accept or adapt each of the individual recommendations in the latest ESMO Clinical Practice Guidelines.^20^ The 20 voting Asian experts were asked to vote YES or NO (one vote per society) on the ‘acceptability’ (agreement with the scientific content of the recommendation) and ‘applicability’ (availability, reimbursement and practical challenges) of each of the ESMO recommendations in a pre-meeting survey (see Methodology in Supplementary Material S1, available at https://doi.org/10.1016/j.esmoop.2022.100744). For recommendations, where a consensus was not reached, the Asian experts were invited to modify the wording of the recommendation(s) at the virtual ‘face-to-face’ meeting using further rounds of voting, if necessary, in order to determine the definitive acceptance or rejection of an adapted recommendation and discuss the applicability challenges. The ‘Infectious Diseases Society of America-United States Public Health Service Grading System’ (Supplementary Material S4, available at https://doi.org/10.1016/j.esmoop.2022.100744)^21^ was used to define the LoE and strength (grade) of each recommendation. Any modifications to the initial recommendations were highlighted in bold text in a summary table of the final Asian recommendations and in the main text, if applicable. A consensus was considered to have been achieved when ≥80% of experts voted that a recommendation was acceptable.

## Results

In the initial pre-meeting survey, the 20 voting Asian experts reported on the ‘acceptability’ and ‘applicability’ of the 51 recommendations for the diagnosis, treatment and follow-up of patients with endometrial cancer from the 2022 ESMO Clinical Practice Guidelines.^20^ These recommendations were made in the five categories outlined in the text below and in Table 1.

**Table 1:** Summary of Asian recommendations for the treatment of patients with endometrial cancer

Recommendations	Acceptability consensus
Recommendation 1: Diagnosis, pathology and molecular biology	
1a. Histological type, FIGO grade, myometrial invasion and LVSI (focal/substantial) should be described for all endometrial cancer pathology specimens [V, A]. 1b. Molecular classification through well-established IHC staining for p53 and MMR proteins (MLH1, PMS2, MSH2, MSH6) in combination with targeted tumour sequencing (POLE hotspot analysis) should be carried out for all endometrial cancer pathology specimens regardless of histological type [IV, A].	100% 100%
Recommendation 2: Staging and risk assessment	
2a. Obtaining endometrial sampling by biopsy or dilatation and curettage (D & C) are acceptable initial approaches to the histological diagnosis of endometrial cancer [IV, A]. 2b. The preoperative work-up should include clinical and gynaecological examination, transvaginal ultrasound, pelvic MRI, a full blood count and liver and renal function profiles [IV, B]. 2c. Additional imaging tests (e.g. abdominal CT and thoracic scan and/or FDG–PET–CT) may be considered in those patients at high risk of extra-pelvic disease [IV, C].	100% 100% 100%
Recommendation 3: Management of local and locoregional disease	
Surgery 3a. Hysterectomy with bilateral salpingo-oophorectomy is the standard surgical procedure in early-stage endometrial cancer [I, A]. 3b. Minimally invasive surgery is the recommended approach in stage I G1-G2 endometrial cancer [I, A]. 3c. Minimally invasive surgery may also be the preferred surgical approach in stage I G3 [II, A]. 3d. Ovarian preservation can be considered in premenopausal women with stage IA G1 endometrioid-type endometrial cancer [IV, A]. 3e. Sentinel lymph node excision (SLNE) can be considered as a strategy for nodal assessment in cases of low-risk/intermediate-risk endometrial cancer (e.g. stage IA, G1-G3 and stage IB, G1-G2) **in experienced centres** [II, A]. It can be omitted in cases without myometrial invasion. **When SLNE is not available, LNE can be carried out in patients with stage 1A G3 and stage 1B disease [II, B].** 3f. Surgical lymph node staging should be carried out in patients with high-intermediate-risk/high-risk disease. Sentinel lymph node biopsy is an acceptable alternative to systematic LNE for lymph node staging in high-intermediate/high-risk stage I-II endometrial cancer **when available and in centres with experience** [III, B]. 3g. Full surgical staging including omentectomy, peritoneal biopsies and lymph node staging should be considered in serous endometrial cancers and carcinosarcomas [IV, B]. 3h. When feasible, and with acceptable morbidity, cytoreductive surgery to the maximal surgical extent should be considered in patients with stage III and IV disease [IV, B].	100% 100% 100% 100% 100% 100% 100% 100%
Low-risk endometrial cancer	
3i. For patients with stage IA (G1 and G2) endometrioid (dMMR and NSMP) type endometrial cancer with no or focal LVSI, adjuvant treatment is not recommended [I, E]. 3j. For patients with stage IA non-endometrioid type (and/or p53-abn), without myometrial invasion and no or focal LVSI, **there are not enough data to make a definitive recommendation regarding adjuvant treatment. Adjuvant therapy (chemotherapy and/or brachytherapy) or no adjuvant treatment may be discussed on a case-by-case basis in a multidisciplinary team approach** [IV, **C**]. 3k. For patients with stage I-II *POLE*mut cancers, **omission of** adjuvant treatment **should be considered** [III, D]. 3l. For patients with stage III *POLE*mut cancers, **there is insufficient evidence on need for adjuvant treatment. Enrolment in** clinical trials**, adjuvant therapy or no adjuvant therapy are reasonable options** [III, C].	100% 100% 100% 100%
Intermediate-risk endometrial cancer	
3m. For patients with stage IA G3 endometrioid (dMMR or NSMP)-type endometrial cancer and no or focal LVSI, adjuvant VBT is recommended to decrease vaginal recurrence [I, A]. 3n. For patients with stage IB G1-G2 endometrioid (dMMR or NSMP)-type endometrial cancer and no or focal LVSI, adjuvant VBT is recommended to decrease vaginal recurrence [I, A]. 3o. For patients with stage II G1 endometrioid (dMMR or NSMP)-type endometrial cancer and no or focal LVSI adjuvant VBT is recommended to decrease vaginal recurrence [II, B]. 3p. Omission of adjuvant VBT can be considered (especially for patients aged <60 years) for all above stages, after patient counselling and with appropriate follow-up [III, C].	100% 100% 100% 100%
High-intermediate-risk endometrial cancer with lymph node staging (pN0)	
3q. For patients with stage IA and IB with substantial LVSI, stage IB G3, stage II G1 with substantial LVSI and stage II G2-G3 (dMMR and NSMP): 3q1. Adjuvant EBRT is recommended [I, A]. 3q2. Adding (concomitant and/or sequential) chemotherapy to EBRT could be considered, especially for G3 and/or substantial LVSI [II, C]. 3q3. Adjuvant VBT (instead of EBRT) could be **considered** to decrease vaginal recurrence, especially for those without substantial LVSI [II, B]. 3q4. **Despite evidence of a benefit from adjuvant treatment, its** omission is an option, when close follow-up can be ensured, following shared decision making with the patient [IV, C].	100% 100% 100% 100% 100%
High-intermediate-risk endometrial cancer without lymph node staging	
3r. For patients with stage IA and IB with substantial LVSI, stage IB G3, stage II G1 with substantial LVSI and stage II G2-G3 (dMMR and NSMP): 3r1. Adjuvant EBRT is recommended [I, A]. 3r2. Adding (concomitant and/or sequential) chemotherapy to EBRT could be considered especially for patients with substantial LVSI and G3 disease [II, C]. 3r3. Adjuvant VBT **followed by chemotherapy** could be considered for patients with stage IB G3 disease without substantial LVSI, **if EBRT is not feasible** [**III, C].**	100% 100% 100% 100%
High-risk endometrial cancer	
3s. Adjuvant EBRT with concurrent and adjuvant chemotherapy is recommended [I, A]. 3t. Sequential chemotherapy and RT can be used [I, B]. 3u. Chemotherapy alone is an alternative option [I, B].	100% 100% 100%
Recommendation 4: Recurrent/metastatic disease	
4a. For patients with locoregional recurrence following primary surgery alone, the preferred primary therapy should be EBRT with **or without** VBT, **depending on the site of recurrence** [IV, A]. 4b. Adding systemic therapy to salvage RT could be considered [IV, C]. 4c. For patients with recurrent disease following RT, surgery should be considered only if a complete debulking with acceptable morbidity is anticipated [IV, C]. 4d. Complementary systemic therapy after surgery could be considered [IV, C]. 4e. The standard first-line chemotherapy treatment is carboplatin AUC 5-6 plus paclitaxel 175 mg/m^2^ every 21 days for six cycles [I, A]. 4f. Hormone therapy could be considered as **an option for** front-line systemic therapy for patients with low-grade carcinomas endometrioid histology **with low-**volume disease [III, A]. 4g. Progestins are the recommended agents [II, A]. 4h. Other options for hormonal therapies include AIs, tamoxifen and fulvestrant [III, C]. 4i. There is no standard of care for second-line chemotherapy. Doxorubicin and weekly paclitaxel are considered the most active therapies [IV, C]. 4j. Immune checkpoint blockade monotherapy **should** be considered after platinum-based therapy failure in patients with MSI-H/dMMR endometrial cancer [III, **A**]. 4k. Dostarlimab **can be considered in patients with dMMR or MSI-H recurrent or advanced endometrial cancer after failure of prior platinum-based chemotherapy and** has recently been approved by both the EMA and the FDA for this indication [III, B; ESMO-Magnitude of Clinical Benefit Scale (ESMO-MCBS) v1.1 score: 3]. 4l. Pembrolizumab is FDA approved for the treatment of TMB-H solid tumours (as determined by the FoundationOne CDx assay) that have progressed following prior therapy for endometrial cancer [III, B; ESMO-MCBS v1.1 score: 3; not EMA approved]. 4m. Pembrolizumab with lenvatinib is approved by the EMA for endometrial cancer patients who have failed a previous platinum-based therapy, and who are not candidates for curative surgery or RT. FDA approval is for endometrial cancer patients whose tumours are not dMMR/MSI-H [I, A; ESMO-MCBS v1.1 score: 4].	100% 100% 100% 100% 100% 100% 100% 100% 100% 100% 100% 100% 100%
Recommendation 5: Follow-up, long-term implications and survivorship	
5a. For low-risk endometrial cancer, the proposed surveillance is **at least** every 6 months, with physical and gynaecological examination for the first 2 years and then yearly until 5 years [V, C]. 5b. In the low-risk group, **remote** follow-up can be **integrated in**to hospital-based follow-up [II, B]. 5c. For the high-risk groups, physical and gynaecological examinations are recommended every 3 months for the first 3 years, and then every 6 months until 5 years [V, C]. 5d. A CT scan or PET–CT could be considered in the high-risk group, particularly if node extension was present [V, D]. 5e. Regular exercise, healthy diet and weight management should be promoted with all endometrial cancer survivors [II, B].	100% 100% 100% 100% 100%

Bold text represents changes to the original recommendations adapted to the Asian context.

AI, aromatase inhibitor; AUC, area under the curve; CT, computed tomography; D & C, dilation and curettage; EBRT, external beam radiotherapy; EMA, European Medicines Agency; ESMO-MCBS, European Society for Medical Oncology-Magnitude of Clinical Benefit Scale; FDA, Food and Drug Administration; FDG–PET, [^18^F]2-fluoro-2-deoxy-D-glucose–positron emission tomography; FIGO, International Federation of Gynaecology and Obstetrics; G 1, 2, 3, grade 1, 2, 3; IHC, immunohistochemistry; LNE, lymphadenectomy; LVSI, lymphovascular space invasion; MMR, mismatch repair; MRI, magnetic resonance imaging; MSI-H, microsatellite instability-high; NSMP, no specific molecular profile, POLE, DNA polymerase-epsilon; RT, radiotherapy; SLNE, sentinel lymph node excision; TMB-H, tumour mutation burden-high; VBT, vaginal brachytherapy**.**

During the pre-meeting survey there were 32 voting discrepancies in relation to scientific ‘acceptability’ (Supplementary Table S2, available at https://doi.org/10.1016/j.esmoop.2022.100744; ‘recommendations 3a, 3e, 3f, 3j, 3k, 3l, 3m, 3n, 3o, 3p, 3q2, 3q3, 3q4, 3r1, 3r2, 3r3, 3s, 3t, 3u, 4a, 4b, 4c, 4e, 4f, 4g, 4h, 4i, 4j,4k, 5a, 5b and 5c’), and 37 voting discrepancies in relation to the ‘applicability’ (Supplementary Table S3, available at https://doi.org/10.1016/j.esmoop.2022.100744) across the 10 different Asian societies.

### 1 Diagnosis, pathology and molecular biology—recommendations 1a-b

Endometrial cancer is clinically a very heterogeneous malignancy for which the assignment of histological subtype, grade, disease extension and lymphovascular space invasion (LVSI) has been highly subjective,^20^^,^^22^ impacting on the accurate assessment of an individual patient’s risk of recurrence and metastasis, and therefore management. Furthermore, it has reduced the ability to accurately compare different clinical studies in terms of outcome due to uncertainty over the classification of patient risk.

The traditional histopathological classification of Bokhman identified two types of endometrial cancer, type I [endometrioid, grade 1-2 (G1-2) with a favourable prognosis], ∼70% of cases, and type II (G3 endometrioid and non-endometrioid histologies with a poor prognosis), ∼30% of cases.^23^ There is general agreement, however, that endometrioid tumours should now be classified according to the International Federation of Gynecology and Obstetrics (FIGO) defined criteria,^20^^,^^24^ providing a two-tier grading system with G1 and G2 endometrioid tumours grouped together as low grade, and G3 tumours classified as high grade. Factors traditionally associated with a high risk of recurrent disease include histologic subtype, FIGO G3 histology, myometrial invasion ≥50%, LVSI,^25^, ^26^, ^27^ L1 cell adhesion molecule expression,^28^^,^^29^ lymph node metastases and tumour diameter >2 cm.

However, the heterogeneity of endometrial cancer is due to an array of underlying molecular alterations. The results of The Cancer Genome Atlas (TCGA) analysis^30^ showed that the molecular diversity of endometrial cancer could be stratified into four distinct molecular subgroups (Supplementary Table S4, available at https://doi.org/10.1016/j.esmoop.2022.100744). The four molecular subgroups are: (i) patients with copy number stable, ultra-mutated endometrial cancers characterised by pathogenic variants in the exonuclease domain of DNA polymerase-epsilon (*POLE*), (ii) patients with hyper-mutated endometrial cancer characterised by microsatellite instability (MSI) due to dysfunctional/deficient mismatch repair genes (dMMR), (iii) an MMR-proficient, low somatic copy number aberration (SCNA) subgroup with a low mutational burden and (iv) a high SCNA subgroup with frequent TP53 mutations. Therefore, well-established immunohistochemical (IHC) staining techniques for the detection of p53 and MMR proteins (MLH1, PMS2, MSH2, MSH6) are now recommended as standard practice for all endometrial cancer pathology specimens, regardless of histological type, together with sequencing of the exonuclease domain of *POLE* if available.^17^ Patients presenting with either newly diagnosed or recurrent/metastatic endometrial cancer should have a biopsy to confirm histology and assess tumour molecular biology.

These molecular classes are identified across all of the histological subtypes,^31^^,^^32^ and correlate with endometrial cancer prognosis.^33^ Thus, molecular classification could facilitate more accurate comparison of clinical outcomes between different groups of patients. Furthermore, it could impact treatment considerations. Firstly, testing for MMR/MSI status serves not only as a screening test for Lynch syndrome, but also identifies patients with metastatic disease who could benefit from immune checkpoint blockade agent. Secondly, the benefit of adjuvant chemotherapy is observed in patients with *p53*mut endometrial cancer,^34^ whilst the de-escalation of therapy in patients with *POLE* mutated (*POLE*mut) endometrial cancer, which has a favourable outcome, is being investigated. Thirdly, the overexpression/gene amplification of human epidermal growth factor receptor 2 (HER2), which has been demonstrated in 20%-40% of type II non-endometrioid endometrial cancers, supports the use of HER2-targeted therapy in combination with chemotherapy. This combined treatment has also recently been shown to be an effective treatment approach for patients with advanced and recurrent serous endometrial cancer.^35^, ^36^, ^37^, ^38^, ^39^ As a consequence, HER2 testing is now being proposed to guide the management of these patients.^40^^,^^41^

Endometrial cancers that have not been completely molecularly classified should be designated as endometrial cancers not-otherwise-specified and use the histology-based classification system.^42^

**Figure 1:**
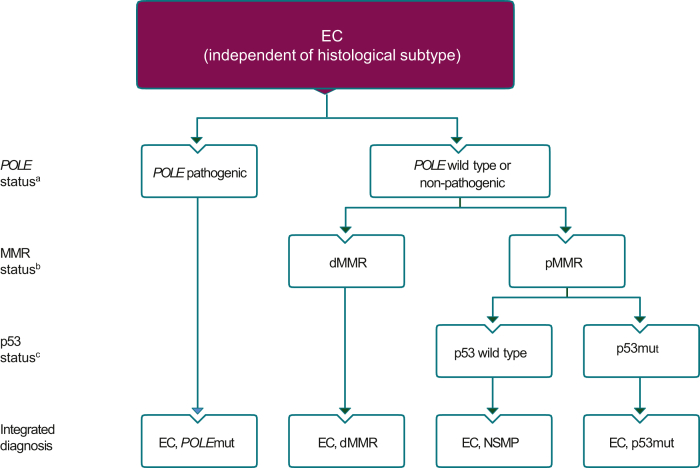
**Diagnostic algorithm for the integrated molecular endometrial cancer classification.** dMMR, mismatch repair deficient; EC, endometrial cancer; MMR, mismatch repair; NSMP, no specific molecular profile; p53mut, p53 mutant; pMMR, mismatch repair proficient; POLE, DNA polymerase epsilon; POLEmut, DNA polymerase epsilon-ultramutated. ^a^Pathogenic POLE variants include p.Pro286Arg, p.Val411Leu, p.Ser297Phe, p.Ala456Pro and p.Ser459Phe.25. ^b^MMR deficiency is defined by the loss of one or more MMR proteins (MLH1, PMS2, MSH2 and MSH6). ^c^p53 immunohistochemistry is an acceptable surrogate marker for TP53 mutation status in MMR-proficient, *POLE* wild-type EC. Permission to use figure under a Creative Commons CC BY License, Wiley obtained by ESMO.

With improved tumour characterisation facilitated by more sophisticated diagnostic testing and molecular profiling, the diagnosis and management of patients with endometrial cancer is evolving towards a more objective, reproducible, personalised medicine approach. The algorithm for the diagnostic work-up of endometrial cancer proposed by ESMO^20^ and adapted from Vermij et al. 2020^42^ is presented in Figure 1.

The Pan-Asian panel of experts agreed with and accepted completely (100% consensus) the ESMO recommendations on diagnosis, pathology and molecular biology ‘recommendations 1a-b’ below and in Table 1. However, they mentioned that *POLE* hotspot mutation analysis was not available as part of the standard molecular evaluation in many centres in Asia.
1a. Histological type, FIGO grade, myometrial invasion and LVSI (focal/substantial) should be described for all endometrial cancer pathology specimens^20^ [V, A].
1b. Molecular classification through well-established IHC staining for p53 and MMR proteins (MLH1, PMS2, MSH2, MSH6) in combination with targeted tumour sequencing (*POLE* hotspot analysis)^43^^,^^44^ should be carried out for all endometrial cancer pathology specimens regardless of histological type^20^ [IV, A].
See Supplementary Material S2, available at https://doi.org/10.1016/j.esmoop.2022.100744, for hereditary endometrial cancer testing and surveillance.

### 2 Staging and risk assessment—recommendations 2a-c

The Pan-Asian panel of experts agreed with and accepted completely (100% consensus) the ESMO recommendations on diagnosis, pathology and molecular biology ‘recommendations 2a-c’ below and in Table 1.^20^

2a. Obtaining endometrial sampling by biopsy or dilation and curettage (D & C) are acceptable initial approaches to the histological diagnosis of endometrial cancer^20^ [IV, A].

2b. The preoperative work-up should include clinical and gynaecological examination, transvaginal ultrasound, pelvic magnetic resonance imaging (MRI),^45^ a full blood count and liver and renal function profiles^20^ [IV, B].

2c. Additional imaging tests [e.g. abdominal and thoracic computed tomography (CT) scan and/or [^18^F]2-fluoro-2-deoxy-D-glucose–positron emission tomography (^18^FDG–PET)–CT may be considered in those patients at high risk of extra-pelvic disease^46^ [IV, C].

### 3 Management of local and locoregional disease—recommendations 3a-u

#### Surgery

Early endometrial cancer is typically treated with surgery to remove the macroscopic disease and stage the tumour for planning with regard to adjuvant therapy.

**Figure 2:**
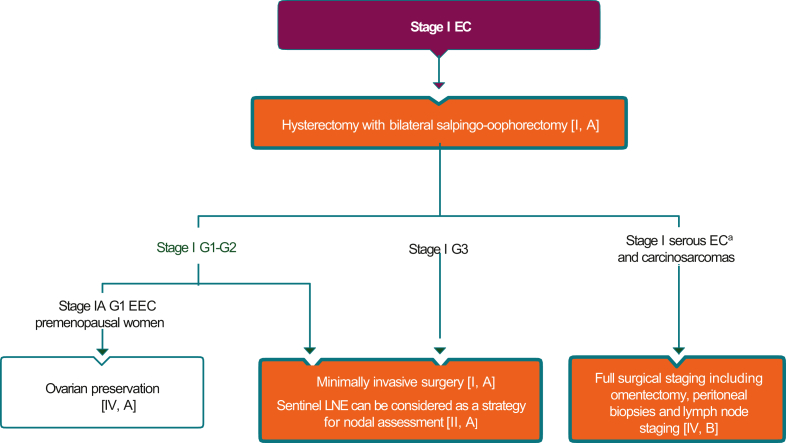
**Stage I endometrial cancer: surgery.** Burgundy box: general category or stratification; orange boxes: surgery; white box: other aspect of management. EC, endometrial cancer; EEC, endometrioid-type endometrial cancer; LNE, lymphadenectomy. ^a^Except in those restricted to polyps.

Traditionally, surgery for endometrial cancer was carried out via laparotomy until the results of two large, randomised trials showed minimally invasive laparoscopic techniques to have no negative impact on either staging or clinical outcomes.^47^^,^^48^ An algorithm for the surgical treatment and management of patients with stage I endometrial cancer is presented in Figure 2. Preservation of fertility in younger patients with endometrial carcinoma should be considered when appropriate^49^ (Supplementary Material S3, available at https://doi.org/10.1016/j.esmoop.2022.100744).

The Pan-Asian panel of experts agreed with and accepted completely (100% consensus) the ESMO recommendations 3a-d below and in Table 1, without change.

3a. Hysterectomy with bilateral salpingo-oophorectomy is the standard surgical procedure in early-stage endometrial cancer [I, A].

3b. Minimally invasive surgery is the recommended approach in stage I (G1-G2) endometrial cancer [I, A] (Figure 2).

3c. Minimally invasive surgery may also be the preferred surgical approach in stage I G3 [II, A] (Figure 2).

3d. Ovarian preservation can be considered in premenopausal women with stage IA, G1 endometrioid-type endometrial cancer [IV, A] (Figure 2).

The comment of the Taiwanese experts with respect to inclusion of sentinel lymph node sampling as part of surgical procedure (recommendation 3a) is covered in recommendation 3e.

However, some Asian experts did not accept ESMO ‘recommendations 3e and 3f’ because they did not reflect real-life clinical practice in their countries with respect to sentinel lymph node excision (SLNE), which is not available in many centres in Asia.

Therefore, the original ‘recommendations 3e and 3f’ were modified, as per the bold text below and in Table 1. However, the consensus was that SLNE should be encouraged wherever possible, based on the evidence available from two studies,^50^^,^^51^ including in patients with deeply invasive endometrioid endometrial cancer,^52^ but not in patients with the more aggressive type II histology^53^^,^^54^ (see ‘recommendation 3g’ below). SLNE can be used for staging in patients with low- or intermediate-risk endometrial cancer and may represent an alternative to systematic lymphadenectomy (LNE) in high-intermediate- or high-risk stage I-II disease.^20^ The randomised Endometrial Cancer Lymphadenectomy Trial (ECLAT) is ongoing in patients with FIGO stage I and II disease with a high risk of recurrence, and should provide more evidence.^55^

3e. SLNE can be considered as a strategy for nodal assessment in cases of low-risk/intermediate-risk endometrial cancer (e.g. stage IA, G1-G3 and stage IB, G1-G2) **in experienced centres** [II, A]. It can be omitted in cases without myometrial invasion. **When SLNE is not available, lymphadenectomy (LNE) can be carried out in patients with stage IA G3 and stage IB disease** [II, B; consensus = 100%].

3f. Surgical lymph node staging should be carried out in patients with high-intermediate-risk/high-risk disease. Sentinel lymph node biopsy is an acceptable alternative to systematic LNE for lymph node staging in patients with high-intermediate/high-risk stage I-II endometrial cancer, **when available and in centres with experience** [III, B; consensus = 100%].

The Pan-Asian panel of experts agreed with and accepted completely (100% consensus) the ESMO ‘recommendations 3g and 3h’ below.

3g. Full surgical staging including omentectomy, peritoneal biopsies and lymph node staging should be considered in serous endometrial cancers and carcinosarcomas [IV, B] (Figure 2).

3h. When feasible, and with acceptable morbidity, cytoreductive surgery to the maximal surgical extent should be considered in patients with stage III and IV disease^20^ [IV, B].

The risk groups for endometrial cancer are summarised in Supplementary Table S5, available at https://doi.org/10.1016/j.esmoop.2022.100744.

#### Low-risk endometrial cancer

There is no indication for the use of adjuvant therapy for the treatment of patients with low-risk endometrial cancer,^56^, ^57^, ^58^ due to a low risk of recurrence. Also, in the few patients in whom local recurrence does occur, it can be treated effectively with radiotherapy (RT). Combined analysis of cohorts from the PORTEC-1 and PORTEC-2 studies^59^ and other studies^33^^,^^60^^,^^61^ has shown the presence of a *POLE* mutation (*POLE*mut) to be a favourable indicator of prognosis, independently of other clinicopathological characteristics. As a consequence, patients with stage I-II endometrial cancer with *POLE*mut tumours are now classified as low risk and unlikely to benefit from adjuvant therapy. Omitting adjuvant therapy in patients with G3 *POLE*mut endometrial cancer may also be an option, although currently there are no robust data available. Higher-level evidence from a prospective registry study is likely to be available shortly together with data from a cohort of the RAINBO trial (NCT05255653). The planned cohorts for the Trans PORTEC RAINBO programme of clinical trials aim to refine the adjuvant treatment of patients with endometrial cancer based on molecular profile including *POLE*mut status, dMMR, no specific molecular profile (NSMP) and abnormal p53 (p53abn).

The Pan-Asian panel of experts agreed with and accepted completely (100% consensus) the ESMO ‘recommendation 3i’ below.

3i. For patients with stage IA (G1 and G2) endometrioid (dMMR and NSMP) type endometrial cancer with no or focal LVSI, adjuvant treatment is not recommended [I, E].

However, some of the Asian experts did not accept the ESMO ‘recommendations 3j, 3k and 3l’, which suggest the omission of adjuvant treatment, because there are little supporting data on the safety of omitting therapy. However, in relation to ‘recommendation 3k’ for patients with stage I-II *POLE*mut disease, there is encouraging, although limited, evidence regarding the omission of adjuvant therapy.^34^^,^^43^

When the *POLE*mut status of a tumour is unavailable, patients should be treated on the basis of the other available risk information. The current focus is on de-escalation of therapy in these patients, whenever possible. Thus, the wording of the original ‘recommendations 3j, 3k and 3l’ (Supplementary Table S2, available at https://doi.org/10.1016/j.esmoop.2022.100744) was revised, as per the bold text below and in Table 1 to reflect the concerns of the Asian experts, with 100% consensus.

3j. For patients with stage IA non-endometrioid-type endometrial cancer (and/or p53abn), without myometrial invasion and no or focal LVSI, **there are not enough data to make a definitive recommendation regarding adjuvant treatment. Adjuvant therapy (chemotherapy and/or brachytherapy) or no adjuvant treatment may be discussed on a case-by-case basis in a multidisciplinary team environment** [IV, C; consensus = 100%].

3k. For patients with stage I-II *POLE*mut cancers, **omission of** adjuvant treatment **should be considered** [III, D; consensus = 100%].

3l. For patients with stage III *POLE*mut cancers, **there is insufficient evidence on need for adjuvant treatment. Enrolment in** clinical trials**, adjuvant therapy or no adjuvant therapy are reasonable options** [III, C; consensus = 100%].

**Figure 3:**
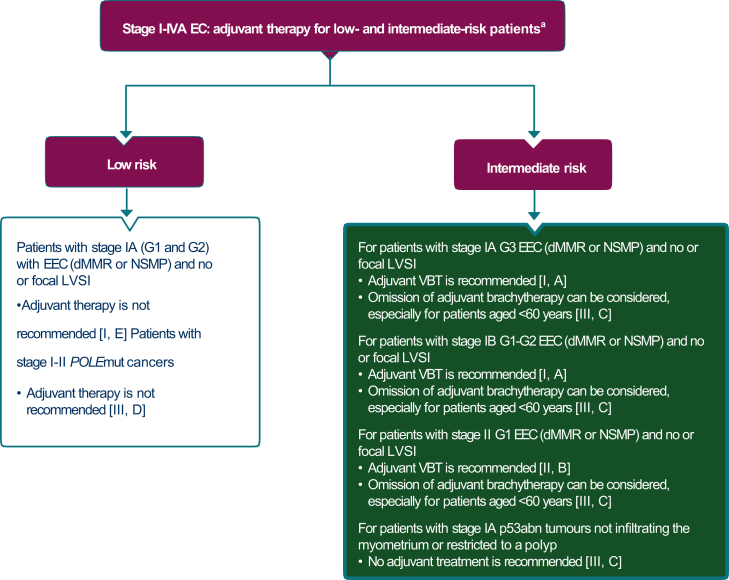
**Stage I-IVA endometrial cancer: adjuvant therapy for low- and intermediate-risk patients.** Burgundy boxes: general categories or stratification; green box: radiotherapy; white box: other aspects of management. dMMR, mismatch repair deficient; EC, endometrial cancer; EEC, endometrioid-type endometrial cancer; LVSI, lymphovascular space invasion; NSMP, no specific molecular profile; p53abn, p53 abnormal; POLEmut, polymerase epsilon-ultramutated; VBT, vaginal brachytherapy. ^a^If completely resected without residual disease.

The adjuvant therapy options for low-risk disease are outlined in Figure 3.

#### Intermediate-risk endometrial cancer

The PORTEC-1^56^ and Gynaecology Oncology Group (GOG)-99^57^ trials demonstrated the benefit of pelvic external beam RT (EBRT) after surgery in reducing locoregional recurrence in patients with intermediate-risk endometrial cancer. However, a Norwegian trial^62^ and an ASTEC study group trial^58^ showed that EBRT and vaginal brachytherapy (VBT) achieve similar results. The long-term results of the PORTEC-2 study showed VBT to result in excellent vaginal control in women with high-intermediate-risk endometrial cancer, with 10-year vaginal control above 96% in both arms. Although the risk of pelvic recurrence was significantly higher in the VBT group (6% versus 1%), no differences were found in 10-year rates for distant metastasis and overall survival. There were lower toxicity rates and better health-related quality of life among women who received VBT compared with EBRT.^63^

The Pan-Asian panel of experts agreed with and accepted completely (100% consensus) the ESMO ‘recommendations 3m, 3n and 3o’ below without change, after much discussion over the use of adjuvant RT. Adjuvant RT is not commonly used in Japan (Supplementary Table S2, available at https://doi.org/10.1016/j.esmoop.2022.100744), with chemotherapy being used as an alternative based on a study by the Japanese Gynecologic Oncology Group.^64^ The experts from China and Taiwan favoured EBRT ± VBT or EBRT alone, respectively, over VBT for stage II G1 endometrial cancer ‘recommendation 3o’.

3m. For patients with stage IA G3 endometrioid (dMMR or NSMP)-type endometrial cancer and no or focal LVSI, adjuvant VBT is recommended to decrease vaginal recurrence [1, A; consensus = 100%].

3n. For patients with stage IB G1-G2 endometrioid (dMMR or NSMP)-type endometrial cancer and no or focal LVSI, adjuvant VBT is recommended to decrease vaginal recurrence [I, A; consensus = 100%].

3o. For patients with stage II G1 endometrioid (dMMR or NSMP)-type endometrial cancer and no or focal LVSI adjuvant VBT is recommended to decrease vaginal recurrence [II, B; consensus = 100%].

It was mentioned by the experts that molecular profiling was not available in certain regions of Asia. In such situations, patients should be treated according to their assessed risk of recurrence.

The Pan-Asian panel of experts agreed with and accepted completely (100% consensus) ‘recommendation 3p’ below without any change.

3p. Omission of adjuvant VBT can be considered (especially for patients aged <60 years) for all above stages, after patient counselling and with appropriate follow-up [III, C].

#### High-intermediate-risk endometrial cancer with lymph node staging (pN0)

There was much discussion over the adjuvant treatment of this group of patients which includes those with stage IA and IB disease with substantial LVSI, stage IB G3 and stage II G1 disease with substantial LVSI and stage II G2-G3 (dMMR or NSMP) disease.

The Pan-Asian panel of experts agreed with and accepted completely (100% consensus) the ESMO ‘recommendation 3q.1’ below, with the proposal from Taiwan that chemotherapy might be considered as an alternative.

3q.1. Adjuvant EBRT is recommended [I, A].

However, some of the Asian experts did not accept the ESMO ‘recommendations 3q.2, 3q.3 and 3q.4’, regarding adjuvant treatment.

With regard to ‘recommendation 3q.2’, some of the experts considered that stronger evidence was needed for the benefit of the addition of chemotherapy, but accepted the recommendation without change based on the data from the PORTEC-3 trial.^65^ However, it was felt that the high incidence of short- and long-term side-effects associated with the addition of chemotherapy to EBRT, whilst conferring minimal benefit, needed to be discussed with these patients.

3q.2. Adding (concomitant and/or sequential) chemotherapy to EBRT could be considered, especially for G3 and/or substantial LVSI [II, C; consensus = 100%].

With regard to ‘recommendation 3q.3’, some of the experts considered that there was insufficient evidence to use the presence or absence of LVSI to decide the type of RT (VBT versus EBRT). In Korea EBRT is used for G3 disease, except in those without LVSI. ‘Recommendation 3q.3’ was accepted completely by replacing ‘could be **recommended**’ with ‘could be **considered**’ as per the bold text below.

3q.3. Adjuvant VBT (instead of EBRT) could be **considered** to decrease vaginal recurrence, especially for those without substantial LVSI [II, B; consensus = 100%].

With regard to ‘recommendation 3q.4’, experts from 6 of the 10 Asian countries considered that adjuvant treatment should be recommended. Thus, the consensus was that the standard treatment for most patients should include adjuvant treatment. However, in highly selected patients (stage IA G1-G2), when close follow-up (every 3 months) is possible, adjuvant treatment may be withheld in consultation with the patient.

Thus, the original ‘recommendation 3q.4’ was revised from:

3q.4. With close follow-up, omission of any adjuvant treatment is an option following shared decision making with the patient [IV, C], to read as the ‘recommendation 3q.4’ below with the new text highlighted in bold.

3q.4. **Despite evidence of a benefit from adjuvant treatment**, **its** omission is an option, when close follow-up can be ensured, following shared decision making with the patient [IV, C].

**Figure 4:**
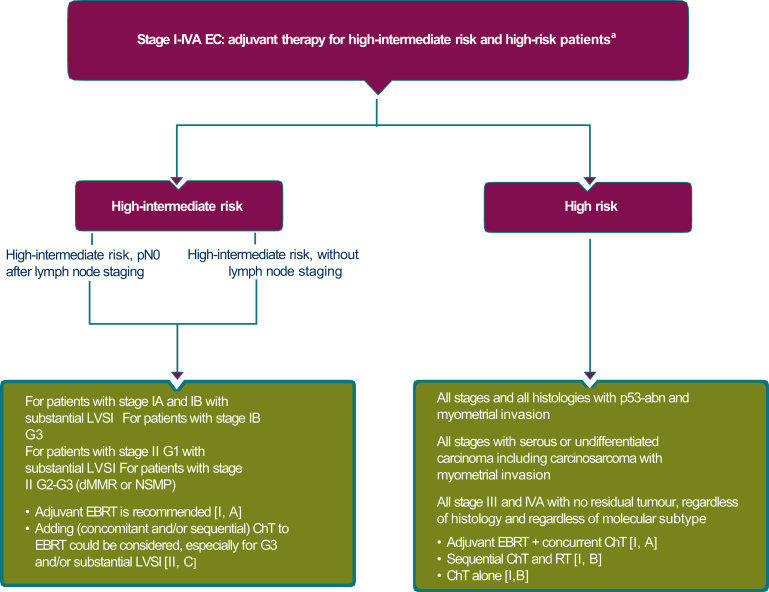
**Stage I-IVA endometrial cancer: adjuvant therapy for high-intermediate-risk and high-risk patients.** Burgundy boxes: general categories or stratification; olive green boxes: combination of treatments or other systemic treatments. ChT, chemotherapy; dMMR, mismatch repair deficient; EBRT, external beam radiotherapy; EC, endometrial cancer; LVSI, lymphovascular space invasion; NSMP, no specific molecular profile; p53abn, p53 abnormal; RT, radiotherapy. ^a^If completely resected without residual disease.

An algorithm for the treatment of these patients is presented in Figure 4.

#### High-intermediate-risk endometrial cancer without lymph node staging

Again, there was much discussion over the adjuvant treatment of this group of patients which includes those with stage IA and IB disease with substantial LVSI, stage IB G3 and stage II G1 disease with substantial LVSI and stage II G2-G3 (dMMR or NSMP) disease.

The Pan-Asian panel of experts agreed with and accepted completely (100% consensus) the ESMO ‘recommendations 3r.1’ below without change.

3r.1. Adjuvant EBRT is recommended [I, A].

With regard to ‘recommendation 3r.2’, experts from some Asian countries, despite the evidence from the PORTEC-1 trial^56^ in patients who had undergone primary surgery (without node dissection) and the PORTEC-3 trial,^66^ were of the opinion that concomitant treatment should be reserved for medically fit patients, but was the preferred option for patients with substantial LVSI. For patients with no initial lymph node dissection, carrying out a lymph node dissection is also an option, followed by tailored adjuvant treatment. ‘Recommendation 3r.2’ below was accepted without change with consideration to be given to the observations cited above.

3r.2. Adding (concomitant and/or sequential) chemotherapy to EBRT could be considered especially for patients with substantial LVSI and G3 disease [II, C; consensus = 100%].

With regard to ‘recommendation 3r.3’, five of the Asian countries did not agree with the original recommendation, and it was generally accepted that in the absence of lymph node staging, EBRT should be considered. Thus the original ‘recommendation 3r.3’ was revised from:

3r.3. Adjuvant VBT could be considered for IB G3 disease without substantial LVSI to decrease vaginal recurrence [II, B], to read as the ‘recommendation 3r.3’ below with the new text highlighted in bold text and the LoE and GoR changed from II, B to III, C.

3r.3. Adjuvant VBT **followed by chemotherapy** could be considered for patients with IB G3 disease without substantial LVSI, **if EBRT is not feasible** [III, C; consensus = 100%].

This recommendation is based on evidence from a subgroup analysis of the phase III GOG-249 trial of adjuvant pelvic RT versus VBT plus paclitaxel/carboplatin in high-intermediate- and high-risk early-stage endometrial cancer.^67^ Radiological evaluation, if not already carried out, should be done before using this option.

An algorithm for the treatment of these patients is presented in Figure 4.

#### High-risk endometrial cancer

There were differences amongst the Asian experts in terms of ‘acceptability’ with regard to ‘recommendations 3s, 3t and 3u’ (see Supplementary Table S2, available at https://doi.org/10.1016/j.esmoop.2022.100744).

There was much discussion over the adjuvant treatment of this group of patients with some of the experts considering the therapy proposed in ‘recommendation 3s’ below too toxic for patients with endometrial cancer due to their age and comorbidities although there are supporting data from the PORTEC-3 trial^65^^,^^66^ and GOG trial^68^ for the benefits of combining chemotherapy with RT in this patient group. High-risk endometrial cancer patients include those with stage III-IVA cancers without residual disease regardless of histology and regardless of molecular subtype, or stage I-IVA p53abn with myometrial invasion, or non-endometrioid cancers without residual disease with myometrial invasion (see Supplementary Table S5, available at https://doi.org/10.1016/j.esmoop.2022.100744). Carcinosarcomas (metaplastic dedifferentiated endometrial cancers) are also regarded as high risk and are commonly classified as p53abn.

However, the Asian experts decided to accept completely the original ESMO ‘recommendation 3s’ below, without change, provided that patients are properly evaluated based on individual factors for this treatment. For patients with major comorbidities or for whom there is an unambiguous contraindication for chemotherapy, RT alone can be considered.

3s. Adjuvant EBRT with concurrent and adjuvant chemotherapy is recommended [I, A; consensus = 100%].

After discussion, the Asian experts also accepted ‘recommendations 3t and 3u’ without change. Extended field RT can be considered along with EBRT and chemotherapy for patients with para-aortic node disease.

3t. Sequential chemotherapy and RT can be used [I, B; consensus = 100%].

3u. Chemotherapy alone is an alternative option [I, B; consensus = 100%].

However, concern was expressed over the use of chemotherapy alone (‘recommendation 3u’), due to the fact that the data regarding comparable efficacy were inconsistent. Certainly, data from the PORTEC-3 trial^34^ showed the treatment effect to differ between the different molecular subgroups. Poor prognosis patients with p53abn endometrial cancer benefitted significantly from chemoradiotherapy (CRT) regardless of stage and histological subtype, whilst patients with *POL*Emut cancers achieved an excellent benefit with either RT or CRT. No benefit was observed for CRT over RT for patients with dMMR endometrial cancer, whilst a trend for benefit was observed in the NSMP subgroup. An algorithm for the treatment of these patients is presented in Figure 4.

For any patients with endometrial cancer who are medically unfit for surgery, by virtue of severe comorbidities, definitive RT is an option (see Supplementary Material S4, available at https://doi.org/10.1016/j.esmoop.2022.100744).

### 4 Recurrent/metastatic disease—recommendations 4a-m

**Figure 5:**
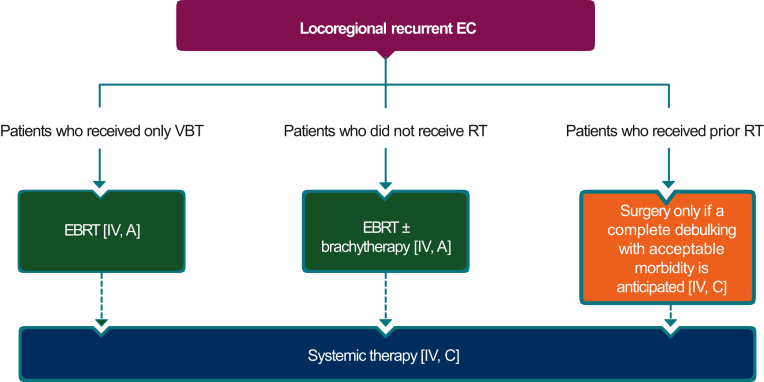
**Locoregional recurrent endometrial cancer.** Burgundy box: general category; orange box: surgery; green boxes: radiotherapy; blue box: systemic anticancer therapy. Dotted arrow denotes optional follow-up therapy. EBRT, external beam radiotherapy; EC, endometrial cancer; RT radiotherapy; VBT, vaginal brachytherapy.

**Figure 6:**
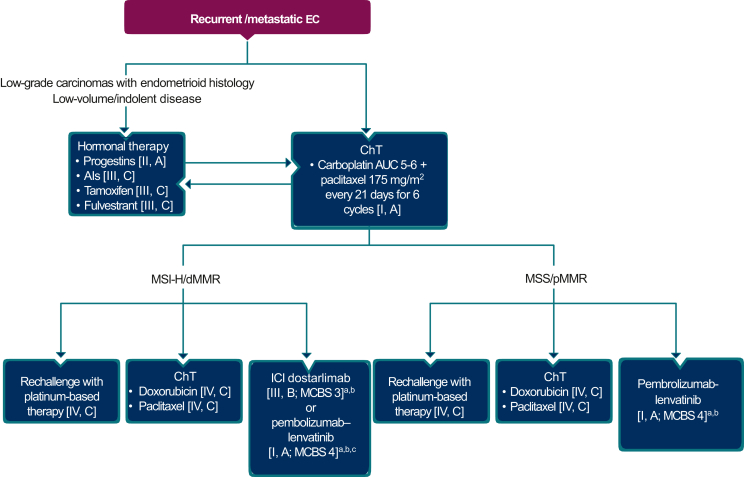
**Metastatic endometrial cancer.** Burgundy box: general category; blue boxes: systemic anticancer therapy. AI, aromatase inhibitor; AUC, area under the curve; ChT, chemotherapy; dMMR, mismatch repair deficient; EC, endometrial cancer; ICI, immune checkpoint inhibitor; ESMO-MCBS, European Society for Medical Oncology-Magnitude of Clinical Benefit Scale; MSI-H, microsatellite instability-high; MSS, microsatellite stable; pMMR, mismatch repair proficient. ^a^In patients eligible for further treatment after failure of platinum-based therapy. ^b^ESMO-MCBS v1.1 was used to calculate scores for new therapies/indications approved by the European Medicines Agency or Food and Drug Administration (FDA). The scores have been calculated by the ESMO-MCBS Working Group and validated by the ESMO Guidelines Committee. ^c^FDA approval is restricted to patients whose tumours are not MSI-H or dMMR.

As stated previously, the outcomes in patients with recurrent and/or metastatic endometrial cancer are poor.^15^ The management of these patients should, wherever possible, involve a multidisciplinary team approach, treatment in specialised centres and the development of individualised treatment plans. Algorithms for the treatment of recurrent locoregional and metastatic disease are presented in Figures 5 and 6, respectively.

Several factors influence the outcomes (local control and survival) in patients with recurrent and/or metastatic disease, including its site and extent (isolated vaginal or peritoneal involvement), size (<2 cm or ≥2 cm), histology and relapse-free survival (RFS). Isolated vaginal recurrence, lower grade, endometrioid histology and longer RFS are associated with a better prognosis.^69^^,^^70^ Additionally, prior treatment (surgery and/or RT) and patient’s general condition also influence outcome.

The Asian experts expressed concern over the omission of surgery from the ESMO ‘recommendation 4a’, and the recommendation of only VBT, which should be considered if there is isolated vaginal recurrence. Thus, ‘recommendation 4a’ was revised by inclusion of the text in bold below.

4a. For patients with locoregional recurrence following primary surgery alone, the preferred primary therapy should be EBRT with **or without** VBT, **depending on the site of recurrence** [IV, A; consensus = 100%].

It was discussed that surgery could be considered in selected patients in whom it is possible to achieve complete surgical resection in the absence of excessive morbidity, and that the use of VBT alone can be considered in the subgroup of patients with a small vaginal recurrence.

‘Recommendations 4b-e’ were accepted without change with the caveat that they may not be applicable in all cases, depending on extent of disease.

4b. Adding systemic therapy to salvage RT could be considered [IV, C; consensus = 100%].

4c. For patients with recurrent disease following RT, surgery should be considered only if a complete debulking with acceptable morbidity is anticipated^71^ [IV, C; consensus = 100%].

4d. Complementary systemic therapy after surgery could be considered^71^, ^72^, ^73^ [IV, C; consensus = 100%] (see Figure 5).

4e. The standard first-line chemotherapy treatment is carboplatin AUC 5-6 plus paclitaxel 175 mg/m^2^ every 21 days for six cycles [I, A; consensus = 100%].

In relation to ‘recommendation 4e’ there is no evidence of an increased benefit for >6 cycles of chemotherapy, but it was agreed that this could be considered on an individual basis.

Some Asian experts did not agree with the original ‘recommendation 4f’ because hormone therapy is rarely offered as first-line systemic therapy in these patients. The experts agreed that chemotherapy is the first choice of treatment. Hormone therapy can be considered for patients with low-grade, low-volume disease who are not suitable for chemotherapy, dependent on knowledge of the hormone receptor status [estrogen receptor (ER) and progesterone receptor (PgR)] of the tumour at the time of treatment. However, the predictive value of hormone receptor expression in endometrial cancer is not as strong as it is for patients with breast cancer due to the limitations associated with a lack of standardisation of tissue processing and factors such as a well-defined cut-off limit in relation to receptor levels.^20^ Furthermore, responses to hormone therapy have been reported in ER-/PgR-negative disease.^74^

Thus, due to these concerns, the text of the original recommendation ‘recommendation 4f’ below was modified by the inclusion of the bold text.

4f. Hormone therapy could be considered as **an option for** front-line systemic therapy **in** patients with low-grade carcinomas of endometrioid histology **with low-volume disease** [III, A; consensus = 100%].

The Asian experts accepted without change ‘recommendations 4g, 4h and 4i’ below, despite some discussion and the removal of the dosing details for medroxyprogesterone acetate and megestrol acetate (Supplementary Table S2, available at https://doi.org/10.1016/j.esmoop.2022.100744) in ‘recommendation 4g’. Aromatase inhibitors and fulvestrant are alternative options with limited benefits.^75^ A phase II study of anastrozole in recurrent ER-/PgR-positive endometrial cancer (the PARAGON trial) showed a low objective response but a meaningful clinical benefit in 44% of patients.^76^

4g. Progestins are the recommended agents [II, A; consensus = 100%].

4h. Other options for hormonal therapies include aromatase inhibitors (AIs), tamoxifen and fulvestrant [III, C; consensus = 100%].

4i. There is no standard of care for second-line chemotherapy. Doxorubicin and weekly paclitaxel are considered the most active therapies^77^, ^78^, ^79^ [IV, C; consensus = 100%].

The Asian experts queried ‘recommendation 4j’, but eventually accepted it without change with the provision that for patients with a long disease-free interval after prior chemotherapy, retreatment with further platinum-based treatment can also be considered, based on a retrospective analysis,^80^ when immune checkpoint inhibitor therapy is not available.

After discussion, the GoR of ‘recommendation 4j’ was revised from B to A [ESCAT IA, ESMO-Magnitude of Clinical Benefit Scale (ESMO-MCBS) 3], as per the bold text below.

4j. Immune checkpoint blockade monotherapy **should** be considered after platinum-based therapy failure in patients with MSI-H/dMMR^81^^,^^82^ [III, A; consensus =100%].

Immune checkpoint blockade alone or in combination with targeted therapies has emerged as a promising intervention in patients with recurrent endometrial cancer in view of a high mutational burden (dMMR/POLEmut subtypes), tumour-infiltrating lymphocytes and programmed cell death protein 1 (PD-1)/programmed death-ligand 1 (PD-L1) expression. Pembrolizumab, which targets PD-1, has been investigated in the endometrial cohorts of the KEYNOTE-158 trial in patients pre-treated with chemotherapy, and a short progression-free survival (PFS), and showed PD-1 blockade to be highly effective.^81^ Data from the GARNET trial with the anti-PD-1 monoclonal antibody dostarlimab, which blocks interaction with the programmed death ligands PD-L1 and -L2, have led to the approval of dostarlimab monotherapy by the Food and Drug Administration (FDA) in the United States to treat dMMR recurrent or advanced endometrial cancer that has progressed on platinum-containing regimens^82^ (Figure 6). Agents that target PD-L1 such as avelumab^83^ and durvalumab^84^ have also shown promising activity in patients with dMMR endometrial cancer, as well as atezolizumab and nivolumab (anti-PD-1).^85^ The phase Ib/II KEYNOTE 146 trial^86^ showed encouraging response, PFS and overall survival rates with the combination of pembrolizumab and the multi-kinase inhibitor lenvatinib, and the phase III KEYNOTE-775 trial^87^ demonstrated the statistically significant PFS (*P* < 0.0001) and overall survival (*P* < 0.0001) benefits of this combination compared with standard chemotherapy. As a consequence, pembrolizumab in combination with lenvatinib has been approved by the FDA for patients with advanced endometrial cancer, that is not MSI-high (MSI-H) or dMMR, who have disease progression following prior systemic therapy in any setting and are not candidates for curative surgery or RT. The European Medicines Agency (EMA) approved pembrolizumab in combination with lenvatinib for the treatment of advanced or recurrent endometrial cancer in patients who have disease progression on or following prior treatment with a platinum-containing regimen in any setting regardless of MMR status and who are not candidates for curative surgery or RT (Figure 6).

However, due to the lack of availability of dostarlimab in 6 of the 10 Asian countries, the wording of the original ‘recommendation 4k’ was reworded from the original ESMO recommendation below,

4k. Dostarlimab has recently been approved by both the EMA and the FDA for this indication^82^ [III, B; ESMO-Magnitude of Clinical Benefit Scale (ESMO-MCBS) v1.1 score: 3],

to read as follows:

4k. Dostarlimab **can be considered in patients with dMMR or MSI-H recurrent or advanced endometrial cancer after failure of prior platinum-based chemotherapy** and has recently been approved by both the EMA and the FDA for this indication [III, B; consensus = 100%; ESMO-Magnitude of Clinical Benefit Scale (ESMO-MCBS) v1.1 score: 3].

The Asian experts accepted completely without change (100% consensus) the original ESMO recommendations ‘recommendations 4l and 4m’ below and in Table 1.

4l. Pembrolizumab is FDA approved for the treatment of TMB-H solid tumours (as determined by the FoundationOne CDx assay) that have progressed following prior therapy for endometrial cancer^88^ [III, B; ESMO-MCBS v1.1 score: 3; not EMA approved].

4m. Pembrolizumab with lenvatinib is approved by the EMA for endometrial cancer patients who have failed a previous platinum-based therapy, and who are not candidates for curative surgery or RT. FDA approval is for endometrial cancer patients whose tumours are not dMMR/MSI-H [I, A; ESMO-MCBS v1.1 score: 4].

Targeted therapy approaches are also being investigated in patients with endometrial cancer. Uterine serous carcinoma (USC) is an aggressive endometrial cancer subtype associated with a poor outcome.^89^ One-third of USCs overexpress HER2/Neu,^35^ a target for trastuzumab in breast cancer. A small randomised phase II trial for the addition of trastuzumab to paclitaxel/carboplatin compared with paclitaxel/carboplatin alone in stage III-IV or recurrent USC demonstrated a meaningful benefit for PFS [hazard ratio (HR) 0.46, *P* = 0.005] and overall survival (HR 0.58).The benefit for stage III-IV was greater than in recurrent disease.^37^ The cyclin-dependent kinase inhibitor palbociclib has shown superiority in combination with letrozole in previously treated patients with ER-positive disease in the phase II ENGOT EN3 PALEO trial,^90^ and the WEE1 inhibitor adavosertib has been investigated in heavily pre-treated patients with serous tumours.^91^ Future directions include immune checkpoint blockade strategies in combination with other targeted therapies, immunotherapeutic agents, chemotherapy and RT.^20^

### 5 Follow-up, long-term implications and survivorship—recommendations 5a-e

There is no evidence from randomised studies to support intensive, clinician-led, hospital-based, follow-up evaluations for patients with endometrial cancer and no consensus on what surveillance tests should be carried out.^20^^,^^92^ Thus, clinical monitoring can be adjusted according to the risk factors of the patient.

There was considerable discussion amongst the Asian experts about the frequency of follow-up appointments with no evidence of a survival benefit from intensive versus minimalist follow-up, even in high-risk patients, as demonstrated by the results of the European multicentre phase III TOTEM trial.^93^ Furthermore, the evidence showed that there was no need to add routine vaginal cytology, laboratory investigations or imaging to the minimalist follow-up strategies.

Thus, ‘recommendation 5a’ was modified very slightly as per the bold text below.

5a. For low-risk endometrial cancer, the proposed surveillance is **at least** every 6 months for the first 2 years and then yearly until 5 years. A physical and gynaecological examination should be performed at each follow-up [V, C; consensus = 100%].

With regard to ‘recommendation 5b’ the experts were concerned that access to phone follow-up would be difficult in certain regions. Therefore, ‘recommendation 5b’ (Supplementary Table S2, available at https://doi.org/10.1016/j.esmoop.2022.100744) was reworded to:

5b. In the low-risk group, **remote** follow-up can be **integrated in**to hospital-based follow-up [II, B; consensus = 100%].

The Asian experts accepted ‘recommendations 5c, d and e’ below without change despite concern over the frequency/timing of follow-up in ‘recommendation 5c’.

5c. For the high-risk groups, physical and gynaecological examinations are recommended every 3 months for the first 3 years, and then every 6 months until 5 years [V, C].

5d. A CT scan or PET–CT could be considered in the high-risk group, particularly if node extension was present [V, D].

5e. Regular exercise, healthy diet and weight management should be promoted with all endometrial cancer survivors [II, B].

### Availability of diagnostic tests, drugs and equipment

Following the virtual face-to-face meeting hosted by ISMPO, the Pan-Asian panel of experts agreed with and accepted completely (100% consensus) the adapted ESMO guidelines listed in Table 1.

The drug and treatment availability for each of the 10 Asian countries is summarised in Supplementary Table S6, available at https://doi.org/10.1016/j.esmoop.2022.100744, and the ESMO-MCBSs for the different systemic therapy options and new therapy combinations for the treatment of endometrial cancer are presented in Supplementary Table S7, available at https://doi.org/10.1016/j.esmoop.2022.100744, and %%=%%=+%%=+https://www.esmo.org/guidelines/esmo-mcbs/esmo-mcbs-scorecards?mcbs_score_cards_form5BsearchText5D%mcbs_score_cards_form%5Btumour-type%5D=Gynaecological+Malignancies&mcbs_score_cards_form%5Btumour-sub-type%5D=Endometrial+Cancer. There was only one area of discrepancy in terms of diagnostic tests, drugs and equipment. This was *POLE* hotspot mutation analysis and the lack of/limited availability of such analysis in five of the Asian countries represented at the meeting.

### Conclusions

The results of voting by the Asian experts before (Supplementary Table S2, available at https://doi.org/10.1016/j.esmoop.2022.100744) and after the virtual/face-to-face working meeting showed >80% concordance (Table 1) with the ESMO recommendations for the treatment of patients with endometrial cancer. Following the virtual ‘face-to-face’ discussions, revisions were made to the wording of ‘recommendations 3e, 3f, 3j, 3l, 3q.4, 3r.3, 4a, 4k and 5b’ (Table 1), and resulted in the achievement of 100% consensus for all the recommendations listed in Table 1.

Thus, the recommendations detailed in Table 1 can be considered the consensus clinical practice guidelines for the treatment of patients with endometrial cancer in Asia. As mentioned previously, the acceptance of each recommendation by each of the Asian experts was based on the available scientific evidence and was independent of the approval and reimbursement status of certain procedures and drugs in the individual Asian countries. A summary of the availability of the recommended treatment modalities and recommended drugs, as of July 2022, is presented for each participating Asian country in Supplementary Table S6, available at https://doi.org/10.1016/j.esmoop.2022.100744, and will impact on some management strategies that can be adopted by certain Asian countries.
