# Supplementary material for: Pan-Asian adapted ESMO Clinical Practice Guidelines for the diagnosis, treatment and follow-up of patients with endometrial cancer
Source: ESMO Open. 2023 Jan 23;8(1):100774. doi: 10.1016/j.esmoop.2022.100774 (PMC10024150; doi:10.1016/j.esmoop.2022.100774)
Supplement: Supplementary material 1 [file mmc1.docx]

**Supplementary material:**

**Pan-Asian adapted ESMO Clinical Practice Guidelines for the diagnosis, treatment and follow-up of patients with endometrial cancer**

**1. Methodology**

A set of preformulated recommendations, grouped into eight categories, for the treatment of endometrial cancer, based on those in the latest ESMO Clinical Practice Guidelines^1^ and updates were circulated, prior to the virtual ‘face to face’ meeting, to each of the 20 Asian experts representing the 10 Asian oncology societies to collect their comments in terms of both the *acceptability* of each recommendation from a purely scientific point of view (i.e. independent of the actual access to, and availability of, the respective diagnostic tools and treatments in their respective countries), and the *applicabilit*y of each recommendation (in terms of regulatory approval, availability and reimbursement status), as it related to the situation in their individual countries. Specific emphasis was placed on current agreement with the published data used for the recommendations in their countries, the data generated from studies in Asian patients, together with the applicability of novel clinical study data to current practice in their countries. Most statements were based on peer-reviewed manuscript data or peer-reviewed abstract data, although statements made based on expert opinion were also considered to be justified standard clinical practice by the experts, and the ISMPO and ESMO faculty.

**References**

1 Oaknin A, Bosse TJ, Creutzberg CL et al. Endometrial cancer: ESMO Clinical Practice Guideline for diagnosis, treatment and follow-up. Ann Oncol 2022.
