# Supplementary material for: Pan-Asian adapted ESMO Clinical Practice Guidelines for the diagnosis, treatment and follow-up of patients with endometrial cancer
Source: ESMO Open. 2023 Jan 23;8(1):100774. doi: 10.1016/j.esmoop.2022.100774 (PMC10024150; doi:10.1016/j.esmoop.2022.100774)
Supplement: Supplementary material 2 [file mmc2.docx]

**Supplementary material**

**2. Hereditary endometrial cancer (testing and surveillance)**

1. About 3% of endometrial cancers and 10% of mismatch repair deficient (dMMR) cancers are related to Lynch syndrome due to germline mutations of one of the MLH1, PMS2, MSH2, MSH6 genes or EPCAM.^1,^ ^2^
2. MMR–Immunohistochemistry (IHC) to assess the expression of these proteins on tumour tissue serves as a pre-screen for Lynch Syndrome and is recommended in all patients with endometrial cancer.^3^
3. The molecular analysis of microsatellite instability (MSI) status is an alternative but expensive test.
4. Testing for MLH1 methylation status to exclude a non-hereditary endometrial cancer is needed if there is a loss of MLH1/PMS2 on IHC testing.

The diagnosis of Lynch Syndrome in patients with dMMR or high MSI requires germline mutation testing for the MMR genes. The identified carriers need to be informed of the life-time risk of colorectal cancer (20-70% depending on the genes involved) and require colonoscopies every 1-2 years. and genetic counselling. Germline mutation testing of relatives should be performed to identify carriers who then require surveillance for signs of endometrial cancer annually from age of 35 until hysterectomy.^4^ Total abdominal hysterectomy with bilateral salpingo-oophorectomy (TAH + BSO) should be performed preferably before 40 years of age after completion of the family. Hormone replacement therapy is also recommended post-surgery.

Other rare hereditary syndromes include COWDEN (germline PTEN mutation) and those related to BRCA1/2. mutations.^4^

**References**

1 Smith ES, Da Cruz Paula A, Cadoo KA et al. Endometrial Cancers in BRCA1 or BRCA2 Germline Mutation Carriers: Assessment of Homologous Recombination DNA Repair Defects. JCO Precis Oncol 2019; 3.

2 Ryan NAJ, Glaire MA, Blake D et al. The proportion of endometrial cancers associated with Lynch syndrome: a systematic review of the literature and meta-analysis. Genet Med 2019; 21 (10): 2167-2180.

3 Mills AM, Liou S, Ford JM et al. Lynch syndrome screening should be considered for all patients with newly diagnosed endometrial cancer. Am J Surg Pathol 2014; 38 (11): 1501-1509.

4 Ryan NAJ, Morris J, Green K et al. Association of Mismatch Repair Mutation With Age at Cancer Onset in Lynch Syndrome: Implications for Stratified Surveillance Strategies. JAMA Oncol 2017; 3 (12): 1702-1706.
