# Supplementary material for: Pan-Asian adapted ESMO Clinical Practice Guidelines for the diagnosis, treatment and follow-up of patients with endometrial cancer
Source: ESMO Open. 2023 Jan 23;8(1):100774. doi: 10.1016/j.esmoop.2022.100774 (PMC10024150; doi:10.1016/j.esmoop.2022.100774)
Supplement: Supplementary material 3 [file mmc3.docx]

**Supplementary material:**

**3. Fertility preservation options in patients with endometrial cancer**

Fertility preservation may be considered in carefully selected young and otherwise fertile patients who are desirous for future fertility.^1, 2^ Patients with atypical endometrial hyperplasia or well-differentiated endometrioid adenocarcinoma, disease limited to the endometrium confirmed on MRI (preferred) or on transvaginal ultrasound and without evidence of extra-uterine disease may be offered high dose progesterone therapy after obtaining written and informed consent.^3, 4^ Dilation and curettage (D&C) is preferred over endometrial biopsy, and histopathology should be confirmed by an expert pathologist. Consultation with a fertility expert and genetic evaluation for inherited cancer prior to therapy should be done.

**Recommendations**

1. Continuous, high dose progesterone therapy (megestrol or medroxyprogesterone with or without progestin IUD) is the treatment of choice.
2. In patients with polycystic ovary syndrome, weight management and lifestyle modifications should also be offered along with medical management.
3. Response to treatment should be monitored by imaging and endometrial biopsy every3–4 months. Conception is encouraged in patients who showed complete response to therapy. Patients who have persistent disease at 6-9 months of treatment should undergo hysterectomy. Ovarian preservation may be considered in selected patients.

**References**

1 Greenwald ZR, Huang LN, Wissing MD et al. Does hormonal therapy for fertility preservation affect the survival of young women with early-stage endometrial cancer? Cancer 2017; 123 (9): 1545-1554.

2 Gunderson CC, Fader AN, Carson KA et al. Oncologic and reproductive outcomes with progestin therapy in women with endometrial hyperplasia and grade 1 adenocarcinoma: a systematic review. Gynecol Oncol 2012; 125 (2): 477-482.

3 Ramirez PT, Frumovitz M, Bodurka DC et al. Hormonal therapy for the management of grade 1 endometrial adenocarcinoma: a literature review. Gynecol Oncol 2004; 95 (1): 133-138.

4 Ushijima K, Yahata H, Yoshikawa H et al. Multicenter phase II study of fertility-sparing treatment with medroxyprogesterone acetate for endometrial carcinoma and atypical hyperplasia in young women. J Clin Oncol 2007; 25 (19): 2798-2803.
