# Supplementary material for: Pan-Asian adapted ESMO Clinical Practice Guidelines for the diagnosis, treatment and follow-up of patients with endometrial cancer
Source: ESMO Open. 2023 Jan 23;8(1):100774. doi: 10.1016/j.esmoop.2022.100774 (PMC10024150; doi:10.1016/j.esmoop.2022.100774)
Supplement: Supplementary material 4 [file mmc4.docx]

**Supplementary material**

**4.Treatment of endometrial cancer in medically unfit patients**

Definitive radiation therapy (RT) can be considered for endometrial cancer in patients who are medically unfit by virtue of severe co-morbidities to undergo surgery.^1,2^ Definitive RT with brachytherapy (BT), external beam RT (EBRT) or a combination of both modalities can be considered.^3^ Imaging should be performed to assess the extent of locoregional disease and to rule out distant metastases. EBRT or BT should be image guided when available.

**Recommendations**

1. For low-grade and/or superficial tumours, brachytherapy alone can be considered (II, B).
2. For high-grade tumours and/or deep myometrial invasion, combination of EBRT and brachytherapy should be used (II, B).

**References:**

1. van der Steen-Banasik E, Christiaens M, Shash E, *et al*. Systemic review: radiation therapy alone in medical non-operable endometrial carcinoma. Eur J Cancer 2016;65:172–81.

2. Dutta SW, Trifiletti DM, Grover S, *et al*. Management of elderly patients with early-stage medically inoperable endometrial cancer: systematic review and National Cancer Database analysis. Brachytherapy 2017;16:526–33.

3. Schwarz JK, Beriwal S, Esthappan J, *et al*. Consensus statement for brachytherapy for the treatment of medically inoperable endometrial cancer. Brachytherapy 2015;14:587–99.
