# Supplementary material for: Pan-Asian adapted ESMO Clinical Practice Guidelines for the diagnosis, treatment and follow-up of patients with endometrial cancer
Source: ESMO Open. 2023 Jan 23;8(1):100774. doi: 10.1016/j.esmoop.2022.100774 (PMC10024150; doi:10.1016/j.esmoop.2022.100774)
Supplement: Supplementary Table S1 [file mmc5.docx]

**Supplementary Table S 1.** Levels of evidence and grades of recommendation used by the panel of Asian experts in evaluating the ESMO consensus guidelines for the management of endometrial cancer (adapted from the Infectious Diseases Society of America-United States Public Health Grading system^a^)^1^

| **Levels of evidence** | |
| --- | --- |
| I | Evidence from at least one large randomised, controlled trial of good methodological quality (low potential for bias) or meta-analyses of well-conducted randomised trials without heterogeneity |
| II | Small, randomised trials or large randomised trials with a suspicion of bias (low methodological quality) or meta-analyses of such trials or of trials with demonstrated heterogeneity |
| III | Prospective cohort studies |
| IV | Retrospective cohort studies of case-control studies |
| V | Studies without control group, case reports, expert opinions |
| **Grades of recommendation** | |
| A | Strong evidence for efficacy with a substantial clinical benefit, strongly recommended |
| B | Strong or moderate evidence for efficacy but with a limited clinical benefit, generally recommended |
| C | Insufficient evidence for efficacy or benefit does not outweigh the risk or the disadvantages (adverse events, costs, …) optional |
| D | Moderate evidence against efficacy or for adverse outcome, generally not recommended |
| E | Strong evidence against efficacy or for adverse outcome, never recommended |

^a^By kind permission of Oxford University Press on behalf of the Infectious Diseases Society of America^1^

**Reference**

1 Dykewicz CA, Centers for Disease C, Prevention et al. Summary of the guidelines for preventing opportunistic infections among hematopoietic stem cell transplant recipients. Infect Dis. 2001;33(2):139-144.
