# Supplementary material for: Pan-Asian adapted ESMO Clinical Practice Guidelines for the diagnosis, treatment and follow-up of patients with endometrial cancer
Source: ESMO Open. 2023 Jan 23;8(1):100774. doi: 10.1016/j.esmoop.2022.100774 (PMC10024150; doi:10.1016/j.esmoop.2022.100774)
Supplement: Supplementary Table S2 [file mmc6.docx]

**Supplementary Table S 2.** Results of pre-meeting survey for the *acceptability* of the proposed recommendations for the treatment of endometrial cancer according to Asian oncological society

| **Summary of Asian recommendations** | **CSCO** | **ISMPO** | **ISHMO** | | | **JSMO** | | | | **KSMO** | | | | **MOS** | | | | | **PSMO** | | | | | **SSO** | | | | | | | **TOS** | | | | | | | **TSCO** | | | | | | |
| --- | --- | --- | --- | --- | --- | --- | --- | --- | --- | --- | --- | --- | --- | --- | --- | --- | --- | --- | --- | --- | --- | --- | --- | --- | --- | --- | --- | --- | --- | --- | --- | --- | --- | --- | --- | --- | --- | --- | --- | --- | --- | --- | --- | --- |
| ***Recommendation 1: Diagnosis, pathology and molecular biology*** | | | | | | | | | | | | | | | | | | | | | | | | | | | | | | | | | | | | | | | | | | | | |
| 1a. Histological type, FIGO grade, myometrial invasion and LVSI (focal/substantial) should be described for all ECs pathology specimens [V, A]. | Y | Y | Y | | | Y | | | | Y | | | | Y | | | | | Y | | | | | Y | | | | | | | Y | | | | | | | Y | | | | | | |
| 1b. Molecular classification through well-established IHC staining for p53 and MMR proteins (MLH1, PMS2, MSH2, MSH6) in combination with targeted tumour sequencing (POLE hotspot analysis) should be carried out for all EC pathology specimens regardless of histological type [IV, A]. | Y | Y | Y | | | Y | | | | Y | | | | Y | | | | | Y | | | | | Y | | | | | | | Y | | | | | | | Y | | | | | | |
| ***Recommendation 2: Staging and risk assessment*** |  | | | | | | | | | | | | | | | | | | | | | | | | | | | | | | | | | | | | | | | | | | | |
| 2a. Obtaining endometrial sampling by biopsy or D&C are acceptable initial approaches to histological diagnosis of EC [IV, A]. | Y | Y | Y | | | Y | | | | Y | | | | Y | | | | | Y | | | | | Y | | | | | | | Y | | | | | | | Y | | | | | | |
| 2b. The preoperative work-up should include clinical and gynaecological examination, transvaginal ultrasound, pelvic MRI, a full blood count and liver and renal function profiles [IV, B]. | Y | Y | Y | | | Y | | | | Y | | | | Y | | | | | Y | | | | | Y | | | | | | | Y | | | | | | | Y | | | | | | |
| 2c. Additional imaging tests (e.g. thoracic and abdominal CT scan and/or FDG-PET-CT) may be considered in those patients at high-risk of extra-pelvic disease [IV, C]. | Y | Y | Y | | | Y | | | | Y | | | | Y | | | | | Y | | | | | Y | | | | | | | Y | | | | | | | Y | | | | | | |
| ***Recommendation 3: Management of local and locoregional disease*** | | | | | | | | | | | | | | | | | | | | | | | | | | | | | | | | | | | | | | | | | | | | |
| 3a. Hysterectomy with bilateral salpingo-oophorectomy is the standard surgical procedure in early-stage EC [I, A]. | Y | Y | | Y | | | | Y | | | | | Y | | | | Y | | | | | Y | | | | | | | Y | | | | | | | Y | | | | | | | Y | |
| 3b. Minimally invasive surgery is the recommended approach in stage I G1-G2 EC [I, A]. | Y | Y | | Y | | | | Y | | | | | Y | | | | Y | | | | | Y | | | | | | | N | | | | | | | Y | | | | | | | Y | |
| 3c. Minimally invasive surgery may also be the preferred surgical approach in stage I G3 [II, A]. | Y | Y | | Y | | | | Y | | | | | Y | | | | Y | | | | | Y | | | | | | | Y | | | | | | | Y | | | | | | | Y | |
| 3d. Ovarian preservation can be considered in premenopausal women with stage IA G1 EEC [IV, A]. | Y | Y | | Y | | | | Y | | | | | Y | | | | Y | | | | | Y | | | | | | | Y | | | | | | | Y | | | | | | | Y | |
| 3e. Sentinel LNE can be considered as a strategy for nodal assessment in low-risk/intermediate-risk EC (e.g. stage IA G1-G3 and stage IB G1-G2) [II, A]. It can be omitted in cases without myometrial invasion. Systematic LNE is not recommended in this group [II, D]. | Y | Y | | Y | | | | N | | | | | Y | | | | Y | | | | | Y | | | | | | | Y | | | | | | | N | | | | | | | Y | |
| 3f. Surgical lymph node staging should be carried out in patients with high-intermediate-risk/high-risk disease. Sentinel lymph node biopsy is an acceptable alternative to systematic LNE for lymph node staging in high-intermediate/high-risk stage I-II [III, B]. | Y | Y | | Y | | | | N | | | | | Y | | | | Y | | | | | Y | | | | | | | Y | | | | | | | Y | | | | | | | N | |
| 3g. Full surgical staging including omentectomy, peritoneal biopsies and lymph node staging should be considered in serous ECs and carcinosarcomas [IV, B]. | Y | Y | | Y | | | | Y | | | | | Y | | | | Y | | | | | Y | | | | | | | Y | | | | | | | Y | | | | | | | Y | |
| 3h. When feasible, and with acceptable morbidity, cytoreductive surgery to a maximal surgical extent should be considered in stage III and IV [IV, B]. | Y | Y | | Y | | | | Y | | | | | Y | | | | Y | | | | | Y | | | | | | | Y | | | | | | | Y | | | | | | | Y | |
| *Low-risk endometrial cancer (EC)* | | | | | | | | | | | | | | | | | | | | | | | | | | | | | | | | | | | | | | | | | | | | |
| 3i. For patients with stage IA (G1 and G2) with endometrioid (MMRd and NSMP) type and no or focal LVSI, adjuvant treatment is not recommended [I, E]. | Y | Y | | Y | | | | Y | | | | | Y | | | | Y | | | | | Y | | | | | | Y | | | | | | | Y | | | | | | | Y | | |
| 3j. For patients with stage IA non-endometrioid type (and/or p53-abn), without myometrial invasion and no or focal LVSI, adjuvant treatment is not recommended [III, E]. | N | Y | | Y | | | | N | | | | | N | | | | Y | | | | | N | | | | | | Y | | | | | | | N | | | | | | | N | | |
| 3k. For patients with stage I-II POLEmut cancers adjuvant treatment is not recommended [III, D]. | N | Y | | Y | | | | N | | | | | N | | | | Y | | | | | Y | | | | | | Y | | | | | | | Y | | | | | | | Y | | |
| 3l. For patients with stage III POLEmut cancers, treatment within the scope of clinical trials is recommended but no adjuvant treatment is also an option [III, C]. | N | N | | Y | | | | N | | | | | Y | | | | N | | | | | Y | | | | | | Y | | | | | | | N | | | | | | | Y | | |
| *Intermediate-risk EC* | | | | | | | | | | | | | | | | | | | | | | | | | | | | | | | | | | | | | | | | | | | | |
| 3m. For patients with stage IA G3 endometrioid (MMRd and NSMP) type and no or focal LVSI, adjuvant VBT is recommended to decrease vaginal recurrence [I, A]. | Y | Y | | Y | | | | | N | | | | Y | | | | Y | | | | | Y | | | | | Y | | | | | | | Y | | | | | | | Y | | | |
| 3n. For patients with stage IB G1-G2 endometrioid (MMRd and NSMP) type and no or focal LVSI, adjuvant VBT is recommended to decrease vaginal recurrence [I, A]. | Y | Y | | Y | | | | | N | | | | Y | | | | Y | | | | | Y | | | | | Y | | | | | | | Y | | | | | | | N | | | |
| 3o. For patients with stage II G1 endometrioid cancer (MMRd and NSMP) and no or focal LVSI adjuvant VBT is recommended to decrease vaginal recurrence [II, B]. | N | Y | | Y | | | | | N | | | | Y | | | | Y | | | | | Y | | | | | Y | | | | | | | N | | | | | | | Y | | | |
| 3p. Omission of adjuvant VBT can be considered (especially for patients aged <60 years) for all above stages, after patient counselling and with appropriate follow-up [III, C]. | N | Y | | Y | | | | | Y | | | | Y | | | | Y | | | | | Y | | | | | Y | | | | | | | Y | | | | | | | Y | | | |
| *High-intermediate-risk EC with lymph node staging (pN0)* | | | | | | | | | | | | | | | | | | | | | | | | | | | | | | | | | | | | | | | | | | | | |
| 3q. For patients with stage IA and IB with substantial LVSI, stage IB G3, stage II G1 with substantial LVSI and stage II G2-G3 (MMRd and NSMP): |  |  | | |  | | | |  | | |  | | | |  | | | | |  | | | | |  | | | | | | |  | | | | | | |  | | | | |
| 3q1. Adjuvant EBRT is recommended [I, A]. | Y | Y | | | Y | | | | Y | | | Y | | | | Y | | | | | Y | | | | | Y | | | | | | | Y/N | | | | | | | Y | | | | |
| 3q2. Adding (concomitant and/or sequential) ChT to EBRT could be considered, especially for G3 and/or substantial LVSI [II, C]. | Y | Y | | | Y | | | | N | | | N | | | | N | | | | |  | | | | | Y | | | | | | | Y | | | | | | | Y | | | | |
| 3q3. Adjuvant VBT (instead of EBRT) could be recommended to decrease vaginal recurrence, especially for those without substantial LVSI [II, B]. | Y | Y | | | Y | | | | Y | | | N | | | | Y | | | | | Y | | | | | Y | | | | | | | Y | | | | | | | Y | | | | |
| 3q4. With close follow-up, omission of any adjuvant treatment is an option following shared decision making with the patient [IV, C]. | N | N | | | Y | | | | Y | | | N | | | | Y | | | | | Y | | | | | Y | | | | | | | Y | | | | | | | N | | | | |
| *High-intermediate-risk EC without lymph node staging* | | | | | | | | | | | | | | | | | | | | | | | | | | | | | | | | | | | | | | | | | | | | |
| 3r. For patients with Stage IA and IB with substantial LVSI, stage IB G3, stage II G1 with substantial LVSI and stage II G2-G3 (MMRd and NSMP): |  |  |  | | |  | | | | |  | | | |  | | | | |  | | | | |  | | | | | | |  | | | | | | |  | | | | | |
| 3r1. Adjuvant EBRT is recommended [I, A]. | Y | Y | | | Y | | | N | | | | Y | | | | Y | | | | | Y | | | | | Y | | | | | | | Y | | | | | | | Y | | | | |
| 3r2. Adding (concomitant and/or sequential) ChT to EBRT could be considered especially for substantial LVSI and G3 [II, C]. | Y | Y | | | Y | | | N | | | | N | | | | N | | | | | Y | | | | | Y | | | | | | | Y | | | | | | | Y | | | | |
| 3r3. Adjuvant VBT could be considered for IB G3 without substantial LVSI to decrease vaginal recurrence [II, B]. | N | N | | | Y | | | N | | | | N | | | | Y | | | | | Y | | | | | Y | | | | | | | N | | | | | | | Y | | | | |
| *High-risk EC* | | | | | | | | | | | | | | | | | | | | | | | | | | | | | | | | | | | | | | | | | | | | |
| 3s. Adjuvant EBRT with concurrent and adjuvant ChT is recommended [I, A]. | Y | Y | | | Y | | | N | | | | Y | | | | Y | | | | | Y | | | | | Y | | | | | | | N | | | | | | | N | | | | |
| 3t. Sequential chemotherapy and RT can be used [I, B]. | Y | Y | | | Y | | | Y | | | | Y | | | | Y | | | | | Y | | | | | Y | | | | | | | N | | | | | | | Y | | | | |
| 3u. Chemotherapy alone is an alternative option [I, B]. | N | Y | | | Y | | | Y | | | | Y | | | | Y | | | | | Y | | | | | Y | | | | | | | Y | | | | | | | N | | | | |
| ***Recommendation 4: Recurrent/metastatic disease*** | | | | | | | | | | | | | | | | | | | | | | | | | | | | | | | | | | | | | | | | | | | | |
| 4a. For patients with locoregional recurrence following primary surgery alone, the preferred primary therapy should be RT with VBT [IV, A]. | N | Y | | | Y | | | | Y | | | N | | | | Y | | | | | Y | | | | | Y | | | | | | | N | | | | | | |  | | | | |
| 4b. Adding systemic therapy to salvage RT could be considered [IV, C]. | Y | Y | | | Y | | | | Y | | | N | | | | Y | | | | | Y | | | | | Y | | | | | | | Y | | | | | | | Y | | | | |
| 4c. For patients with recurrent disease following RT, surgery should be considered only if a complete debulking with acceptable morbidity is anticipated [IV, C]. | N | Y | | | Y | | | | Y | | | N | | | | Y | | | | | Y | | | | | Y | | | | | | | Y | | | | | | | Y | | | | |
| 4d. Complementary systemic therapy after surgery could be considered [IV, C]. | Y | Y | | | Y | | | | Y | | | Y | | | | Y | | | | | Y | | | | | Y | | | | | | | Y | | | | | | | Y | | | | |
| 4e. The first-line standard chemotherapy treatment is carboplatin AUC 5-6 plus paclitaxel 175 mg/m2 every 21 days for six cycles [I, A]. | Y | Y | | | Y | | | | Y | | | N | | | | Y | | | | | Y | | | | | Y | | | | | | | Y | | | | | | | Y | | | | |
| 4f. Hormone therapy could be considered as front-line systemic therapy for patients with low-grade carcinomas endometrioid histology [III, A]. | Y | Y | | | Y | | | | Y | | | N | | | | Y | | | | | Y | | | | | Y | | | | | | | Y | | | | | | | Y | | | | |
| 4g. Progestins (medroxyprogesterone acetate 200 mg and megestrol acetate 160 mg) are the recommended agents [II, A]. | Y | Y | | | Y | | | Y | | | | N | | | | Y | | | | | Y | | | | | Y | | | | | | | Y | | | | | | | Y | | | | |
| 4h. Other options for hormonal therapies include AIs, tamoxifen and fulvestrant [III, C]. | Y | Y | | | Y | | | | Y | | | N | | | | Y | | | | | Y | | | | | Y | | | | | | | Y | | | | | | | Y | | | | |
| 4i. There is no standard of care for second-line chemotherapy. Doxorubicin and weekly paclitaxel are considered the most active therapies [IV, C]. | Y | Y | | | Y | | | | Y | | | N | | | | Y | | | | | Y | | | | | Y | | | | | | | Y | | | | | | | Y | | | | |
| 4j. Immune checkpoint blockade (ICB) monotherapy could be considered after platinum-based therapy failure in patients with MSI-H/MMRd EC [III, B]. | Y | Y | | | N | | | | Y | | | Y | | | | Y | | | | | Y | | | | | Y | | | | | | | Y | | | | | | | Y | | | | |
| 4k. Dostarlimab has recently been approved by both the EMA and the FDA for this indication [III, B; ESMO-Magnitude of Clinical Benefit Scale (ESMO-MCBS) v1.1 score: 3]. | Y | Y | | | N | | | | Y | | | Y | | | | Y | | | | | N | | | | | Y | | | | | | | Y | | | | | | | Y | | | | |
| 4l. Pembrolizumab is FDA approved for the treatment of TMB-H solid tumours (as determined by the FoundationOne CDx assay) that have progressed following prior therapy for EC [III, B; ESMO-MCBS v1.1 score: 3; not EMA approved]. | Y | Y | | | Y | | | | Y | | | Y | | | | Y | | | | | Y | | | | | Y | | | | | | | Y | | | | | | | Y | | | | |
| 4m. Pembrolizumab and lenvatinib is approved by the EMA for EC patients who have failed a previous platinum-based therapy, and who are not candidates for curative surgery or RT. FDA approval is for EC patients whose tumours are not MMRd/MSI-H [I, A; ESMO-MCBS v1.1 score: 4]. | Y | Y | | | Y | | | | Y | | | Y | | | | Y | | | | | Y | | | | | Y | | | | | | | Y | | | | | | | Y | | | | |
| ***Recommendation 5: Follow-up, long-term implications and survivorship*** | | | | | | | | | | | | | | | | | | | | | | | | | | | | | | | | | | | | | | | | | | | | |
| 5a. For low-risk EC, the proposed surveillance is every 6 months, with physical and gynaecological examination for the first 2 years and then yearly until 5 years [V, C]. | Y | Y | | Y | | | N | | | | | | N | | | | | Y | | | | | Y | | | | | | | Y | | | | | | | N | | | | | | | Y |
| 5b. In the low-risk group, phone follow-up can be an alternative to hospital-based follow-up consultation [II, B]. | Y | Y | | Y | | | N | | | | | | N | | | | | Y | | | | | Y | | | | | | | Y | | | | | | | N | | | | | | | Y |
| 5c. For the high-risk groups, physical and gynaecological examination are recommended every 3 months for the first 3 years, and then every 6 months until 5 years [V, C]. | Y | Y | | Y | | | N | | | | | | N | | | | | Y | | | | | Y | | | | | | | Y | | | | | | | Y | | | | | | | Y |
| 5d. A CT scan or PET-CT could be considered in the high-risk group, particularly if node extension was present [V, D].5e. | Y | Y | | N | | | Y | | | | | | Y | | | | | Y | | | | | Y | | | | | | | Y | | | | | | | Y | | | | | | | Y |
| 5e. Regular exercise, healthy diet and weight management should be promoted with all EC survivors [II, B]. | Y | Y | | Y | | | Y | | | | | | Y | | | | | Y | | | | | Y | | | | | | | Y | | | | | | | Y | | | | | | | Y |
|  | | | | | | | | | | | | | | | | | | | | | | | | | | | | | | | | | | | | | | | | | | | | |

CSCO, the Chinese Society of Clinical Oncology; ESMO, European Society for Medical Oncology; ISHMO, the Indonesian Society of Haematology and Medical Oncology; SMPO, the Indian Society of Medical and Paediatric Oncology; JSMO, the Japanese Society of Medical Oncology; KSMO, the Korean Society for Medical Oncology; MOS, the Malaysian Oncological Society; PSMO, the Philippine society of Medical Oncology; SSO, the Singapore Society of Oncology; TOS, the Taiwan Oncology Society; TSCO, the Thailand Society of Clinical Oncology

AI, aromatase inhibitor; AUC, area under the curve; ChT, chemotherapy; CT, computed tomography; D&C, dilation and curettage; EBRT, external beam radiotherapy; EC, endometrial cancer; EMA, European Medicines Agency; ESMO MCBS, ESMO-Magnitude of Clinical Benefit Scale; FDA, Federal drug administration; FDG-PET,^18^F-2-fluoro-2-deoxy-D-glucose-positron emission tomography; FIGO, International Federation of Gynaecology and Obstetrics; IHC, immunohistochemistry; LNE, lymphadenectomy; LVSI, lymphovascular space invasion; MMR (d), mismatch repair (deficient); MRI, magnetic resonance imaging; MSI-H, microsatellite instability-high; NSMP, no specific molecular profile; PET, positron emission tomography; POLE, DNA polymerase‐epsilon; SLNE, sentinel lymph node excision; TMB-H, tumour mutation burden-high; RT, radiotherapy; VBT, vaginal brachytherapy.
