# Supplementary material for: Pan-Asian adapted ESMO Clinical Practice Guidelines for the diagnosis, treatment and follow-up of patients with endometrial cancer
Source: ESMO Open. 2023 Jan 23;8(1):100774. doi: 10.1016/j.esmoop.2022.100774 (PMC10024150; doi:10.1016/j.esmoop.2022.100774)
Supplement: Supplementary Table S4 [file mmc8.docx]

**Supplementary Table S 4.** Molecular and clinicopathological features of endometrial cancer molecular subgroups^1^

|  | ***POLE*mut**  (i.e. *POLE* EDM) | **dMMR**  (i.e. MSI) | **NSMP**  (i.e. p53 wt) | **p53aberrant**  (i.e. p53abn, p53mut) |
| --- | --- | --- | --- | --- |
| Prevalence in TCGA cohort | 5%-15% | 25%-30% | 30%-40% | 5%-15% |
| Associated molecular features | >100 mut/Mb, SCNA-very low, MSS | 10-100 mut/Mb, SCNA-low, MSI | <10mut/Mb, SCNA-low, MSS | <10mut/Mb, ScNA-high, MSS |
| Associated histological features | Endometrioid | Endometrioid | Mostly low grade | All histological subtypes |
|  | Often high grade | Often high grade | Notable absence of TILS | Mostly high grade |
|  | Ambiguous morphology | LVSI substantial | Squamous differentiation | High cytonuclear atypia |
|  | Prominent TILS and TLS | Prominent TILS  MELF-type invasion | ER/PR diffuse | Low level of TILS |
| Associated clinical features | Lower BMI | Higher BMI | Higher BMI | Lower BMI |
|  | Early stage (IA-IB) | Lynch syndrome |  | Advanced stage |
|  | Early onset |  |  | Late onset |
| Diagnostic test | NGS/Sanger/Hotspot:  P286R, V411L, S297F, A456P, S459F | MMR-IHC: MLH1, MSH2, MSH6, PMS2  MSI assay |  | p53-IHC  Mutant-like/abnormal staining |
| Prognosis | Excellent | Intermediate | Intermediate  Stage-dependent | Poor |

Adapted from McAlpine et al.,^2^ with permission from John Wiley and Sons.

BMI, body mass index; dMMR, mismatch repair deficient; EDM, exonuclease domain mutation; ER, (o)estrogen receptor; IHC, immunohistochemistry; LVSI, lymphovascular space invasion; MELF, microcystic elongated and fragmented type of invasion; MMR-IHC, mismatch repair immunohistochemistry; MSI, microsatellite instability; MSS, microsatellite stable; mut/Mb, mutations/megabase; NGS, next-generation sequencing; NSMP, no specific molecular profile; p53abn, p53 abnormal; p53mut, p53 mutant; p53-wt, p53-wild type; PgR, progesterone receptor; *POLE*, polymerase epsilon; *POLE*mut, polymerase epsilon-ultramutated; SCNA, somatic copy number alteration; TCGA, The Cancer Genome Atlas; TIL, tumour infiltrating lymphocyte; TLS, tertiary lymphoid structure.

**References**

1 Oaknin A, Bosse TJ, Creutzberg CL et al. Endometrial cancer: ESMO Clinical Practice Guideline for diagnosis, treatment and follow-up. Ann Oncol 2022.

2 McAlpine J, Leon-Castillo A, Bosse T. The rise of a novel classification system for endometrial carcinoma; integration of molecular subclasses. J Pathol 2018; 244 (5): 538-549.
