# Supplementary material for: Pan-Asian adapted ESMO Clinical Practice Guidelines for the diagnosis, treatment and follow-up of patients with endometrial cancer
Source: ESMO Open. 2023 Jan 23;8(1):100774. doi: 10.1016/j.esmoop.2022.100774 (PMC10024150; doi:10.1016/j.esmoop.2022.100774)
Supplement: Supplementary Table S5 [file mmc9.docx]

**Supplementary Table S 5.** Endometrial Cancer Risk groups^1^

| **Risk group** | **Description^a^** |
| --- | --- |
| **Low risk** | Stage IA (G1 and G2) with endometrioid (MMRd^b^ and NSMP) type and no or focal LVSI |
|  | Stage I/II *POLE*mut cancer; for stage III *POLE*mut cancers^c^ |
| **Intermediate risk** | Stage IA G3 with endometrioid type (dMMR and NSMP) and no or focal LVSI |
|  | Stage IA non-endometrioid (serous, clear-cell, undifferentiated carcinoma, carcinosarcoma, mixed) and/or p53-abn cancers without myometrial invasion and no or focal LVSI |
|  | Stage IB (G1-G2) with endometrioid type (dMMR and NSMP) and no or focal LVSI |
|  | Stage II G1 endometrioid cancer (dMMR and NSMP) and no or focal LVSI |
| **High-intermediate risk** | Stage I endometrioid (dMMR and NSMP) any grade and any depth of invasion with substantial LVSI |
|  | Stage IB G3 with endometrioid type (dMMR and NSMP) regardless of LVSI |
|  | Stage II G1 endometrioid cancer (dMMR and NSMP) with substantial LVSI |
|  | Stage II G2-G3 endometrioid cancer (dMMR and NSMP) |
| **High risk** | All stages and all histologies with p53-abn and myometrial invasion |
|  | All stages with serous or undifferentiated carcinoma including carcinosarcoma with myometrial invasion |
|  | All Stage III and IVA with no residual tumour, regardless of histology and regardless of molecular subtype^b^ |

dMMR, mismatch repair deficient; EC, endometrial cancer; G1-G3, grade 1-3; IHC, immunohistochemistry; LVSI, lymphovascular space invasion; MSI-H, microsatellite instabilityhigh/hypermutated; NSMP, no specific molecular profile; p53-abn, p53-abnormal; POLEmut, polymerase epsilon-ultramutated.

^a^Stage III-IVA if completely resected without residual disease; table does not apply to stage III-IVA with residual disease or for stage IV.

^b^dMMR and MSI-H: Both terms identify a similar EC population. Identification of a defective mismatch repair pathway by IHC (i.e. dMMR) or sequencing to determining

microsatellite instability (i.e. MSI-H).

^c^*POLE*mut stage III might be considered as low risk. Currently there are no data regarding safety of omitting adjuvant therapy.

**Reference**

1 Oaknin A, Bosse TJ, Creutzberg CL et al. Endometrial cancer: ESMO Clinical Practice Guideline for diagnosis, treatment and follow-up. Ann Oncol 2022.
