# Supplementary material for: Pan-Asian adapted ESMO Clinical Practice Guidelines for the diagnosis, treatment and follow-up of patients with endometrial cancer
Source: ESMO Open. 2023 Jan 23;8(1):100774. doi: 10.1016/j.esmoop.2022.100774 (PMC10024150; doi:10.1016/j.esmoop.2022.100774)
Supplement: Supplementary Table S6 [file mmc10.docx]

**Supplementary Table S 6.** Summary of applicability (availability) of drugs, equipment and testing for endometrial cancer according to Asian country

| **Diagnostic tests/drugs/equipment** | **CSCO** | **ISMPO** | **ISHMO** | **JSMO** | **KSMO** | **MOS** | **PSMO** | **SSO** | **TOS** | **TSCO** | **Comments (if "N" please explain why)** |
| --- | --- | --- | --- | --- | --- | --- | --- | --- | --- | --- | --- |
|  | **Available? Y/N** | **Available? Y/N** | **Available? Y/N** | **Available? Y/N** | **Available? Y/N** | **Available? Y/N** | **Available? Y/N** | **Available? Y/N** | **Available? Y/N** | **Available? Y/N** |  |
| **IHC for MMR status** | Y | Y | Y | Y | Y | Y | Y | Y | Y | Y |  |
| **P53 IHC** | Y | Y | Y | Y | Y | Y | Y | Y | Y | Y |  |
| ***POLE* hot spot mutation analysis** | Y | N | Y | N | Y | Y | N | Y | N | N | PSMO: We still have no institution offering molecular analysis for POLE mutation  TSCO: Not in routine practice but only in research setting JSMO: POLE hotspot mutation analysis is not reimbursed in Japan. TOS: available on the research lab basis, but is setting up clinical service in 3 months ISMPO: POLE mutation analysis is not available in most centres |
| **TVUS** | Y | Y | Y | Y | Y | Y | Y | Y | Y | Y | SSO: It is available but not in routine practice. |
| **PET/CT** | Y | Y | Y | Y | Y | Y | Y | Y | Y | Y | TOS: National health insurance not reimbursed |
| **LN Sentinel node excision** | Y | Y | Y | Y | Y | Y | Y | Y | Y | Y |  |
| **LN surgical staging** | Y | Y | Y | Y | Y | Y | Y | Y | Y | Y |  |
| **Vaginal brachytherapy** | Y | Y | Y | Y | Y | Y | Y | Y | Y | Y |  |
| **EBRT** | Y | Y | Y | Y | Y | Y | Y | Y | Y | Y |  |
| **Immune checkpoint inhibitor monotherapy** | Y | Y | Y | Y | Y | Y | Y | Y | Y | Y | TSCO: Limited use due to its cost, financial and reimbursement restriction TOS: National health insurance not reimbursed |
| **Pembrolizumab plus lenvatinib** | Y | Y | Y | Y | Y | Y | Y | Y | Y | Y | TSCO: Limited use due to its cost, financial and reimbursement restriction TOS: National health insurance not reimbursed |

CSCO, the Chinese Society of Clinical Oncology; ESMO, European Society for Medical Oncology; ISHMO, the Indonesian Society of Haematology and Medical Oncology; SMPO, the Indian Society of Medical and Paediatric Oncology; JSMO, the Japanese Society of Medical Oncology; KSMO, the Korean Society for Medical Oncology; MOS, the Malaysian Oncological Society; PSMO, the Philippine Society of Medical Oncology; SSO, the Singapore Society of Oncology; TOS, the Taiwan Oncology Society; TSCO, the Thailand Society of Clinical Oncology

CT, computed tomography; EBRT, external beam radiotherapy; IHC, immunohistochemistry; LN, lymph node, MMR, mismatch repair; PET, positron emission tomography; POLE, DNA polymerase‐epsilon; TVUS transvaginal ultrasound
