# Supplementary material for: Pan-Asian adapted ESMO Clinical Practice Guidelines for the diagnosis, treatment and follow-up of patients with endometrial cancer
Source: ESMO Open. 2023 Jan 23;8(1):100774. doi: 10.1016/j.esmoop.2022.100774 (PMC10024150; doi:10.1016/j.esmoop.2022.100774)
Supplement: Supplementary Table S7 [file mmc11.docx]

**Supplementary Table S 7.** ESMO-MCBS table for new therapies/indications in endometrial cancer

| **Therapy** | **Disease setting** | **Trial** | **Control** | **Absolute survival gain** | **HR (95% CI)** | **QoL/toxicity** | **ESMO- MCBS**  **scorea** |
| --- | --- | --- | --- | --- | --- | --- | --- |
| Dostarlimab | Treatment of adult patients with dMMR/MSI-H  recurrent or advanced endometrial cancer that have progressed on or following prior treatment with a platinum-containing regimen | GARNET^1-3^  Phase I  NCT02715284 | Single arm | ORR: 43.5%  Median DoR:  >9 months (NR)  Median PFS:  12.2 months |  |  | 3  (Form 3) |
| Pembrolizumabb | Patients with  unresectable or  metastatic TMB-H  solid tumours that  have progressed  following prior treatment and have no alternative treatment options | KEYNOTE-158^4^  Phase II  NCT02628067 | Single arm | ORR: 29%  Median DoR:  >9 months (NR)  Median PFS:  2.1 months |  |  | 3  (Form 3) |
|  |  |  | cohort study |  |  |  |  |
|  |  |  |  |  |  |  |  |
|  |  |  |  |  |  |  |  |
| Pembrolizumab | Patients with unresectable or metastatic dMMR/MSI-H solid tumours that have progressed following prior treatment and have no alternative treatment options | KEYNOTE- 158^5, 6^  Phase II  NCT02628067 | Single arm cohort study | ORR: 57.1%  Median PFS:  25.7 months  Median DoR:  >9 months (NR) |  | QoL was not a pre-specified endpoint | 3  (Form 3) |
| Pembrolizumab | Patients with advanced or recurrent dMMR/MSI-H endometrial cancer who have disease progression on or following prior treatment with a platinum-containing therapy in any setting and who are not candidates for curative surgery or RT | KEYNOTE- 158^7, 8^  Phase II  NCT02628067 | Single arm cohort study | ORR: 48%  Median PFS:  13.1 months  Median DoR:  >9 months (NR) |  | QoL was not a pre-specified endpoint | 3  (Form 3). Score . |
| Pembrolizumab  + lenvatinibc | Patients with advanced or recurrent endometrial cancer who have progressed following prior platinum-containing therapy in any setting and who are not candidates for curative surgery or RT | KEYNOTE-775^9^  Phase III  NCT03517449 | TPC  (paclitaxel or  doxorubicin)  Median OS:  11.4 months  Median PFS:  3.8 months | OS gain: 6.9  months  PFS gain: 3.4 months | OS HR: 0.62  (0.51-0.75)  PFS HR: 0.56 (0.47-0.66) | No difference  in QoL  between  treatment groups | 4  (Form 2a) |
| Pembrolizumab  + lenvatinibd | Patients with advanced endometrial cancer that is not MSI-H or dMMR, who have disease progression following prior systemic therapy and are not candidates for curative surgery or RT | KEYNOTE- 775^9^  Phase III  NCT03517449 | TPC  (paclitaxel or doxorubicin)  Median OS:  12.0 months  Median PFS:  3.8 months | OS gain: 5.4 months  PFS gain: 2.8 months | OS HR: 0.68  (0.56-0.84)  PFS HR: 0.6  (0.50-0.72) | No difference in QoL between treatment groups | 4  (Form 2a) |

CI, confidence interval; dMMR, mismatch repair deficient; DoR, duration of response; EMA, European Medicines Agency; ESMO- MCBS, ESMO-Magnitude of Clinical Benefit Scale; FDA, Food and Drug Administration; HR, hazard ratio; MSI-H, microsatellite instability-high; NR, not reached; ORR, objective response rate; OS, overall survival; PFS, progression-free survival; pMMR, mismatch repair proficient; QoL, quality of life; RT, radiotherapy; TMB-H, tumour mutational burden-high; TPC, treatment of physician’s choice.

a The scores have been calculated by the ESMO-MCBS Working Group and validated by the ESMO Guidelines Committee. ESMO-MCBS v1.1.^10^ was used to calculate scores for new therapies/indications approved by the EMA or FDA. (https:/[/www.esm](http://www.esmo.org/guidelines/esmo-mcbs/esmo-mcbs-evaluation-forms))o[.org/guidelines/esmo-mcbs/esmo-mcbs-evaluation-forms).](http://www.esmo.org/guidelines/esmo-mcbs/esmo-mcbs-evaluation-forms))

b FDA approved; not EMA approved.

c EMA approval is irrespective of MSI/MMR status and so data shown are for the entire study population.

d FDA approval is restricted to patients whose tumours are not MSI-H or dMMR and so data shown are for the pMMR study population.

**References:**

1 Oaknin A, Gilbert L, Tinker AV et al. Safety and antitumor activity of dostarlimab in patients with advanced or recurrent DNA mismatch repair deficient/microsatellite instability-high (dMMR/MSI-H) or proficient/stable (MMRp/MSS) endometrial cancer: interim results from GARNET-a phase I, single-arm study. J Immunother Cancer 2022; 10 (1).

2 Oaknin A, Tinker AV, Gilbert L et al. Clinical Activity and Safety of the Anti-Programmed Death 1 Monoclonal Antibody Dostarlimab for Patients With Recurrent or Advanced Mismatch Repair-Deficient Endometrial Cancer: A Nonrandomized Phase 1 Clinical Trial. JAMA Oncol 2020.

3 Kristeleit R, Mathews C, Redondo A et al. Patient-reported outcomes in the GARNET trial in patients with advanced or recurrent mismatch repair-deficient/microsatellite instability-high endometrial cancer treated with dostarlimab. Int J Gynecol Cancer 2022.

4 Marabelle A, Fakih M, Lopez J et al. Association of tumour mutational burden with outcomes in patients with advanced solid tumours treated with pembrolizumab: prospective biomarker analysis of the multicohort, open-label, phase 2 KEYNOTE-158 study. Lancet Oncol 2020; 21 (10): 1353-1365.

5 Marabelle A, Le DT, Ascierto PA et al. Efficacy of Pembrolizumab in Patients With Noncolorectal High Microsatellite Instability/Mismatch Repair-Deficient Cancer: Results From the Phase II KEYNOTE-158 Study. J Clin Oncol 2020; 38 (1): 1-10.

6 Maio M, Amonkar MM, Norquist JM et al. Health-related quality of life in patients treated with pembrolizumab for microsatellite instability-high/mismatch repair-deficient advanced solid tumours: Results from the KEYNOTE-158 study. Eur J Cancer 2022; 169: 188-197.

7 O'Malley DM, Bariani GM, Cassier PA et al. Pembrolizumab in Patients With Microsatellite Instability-High Advanced Endometrial Cancer: Results From the KEYNOTE-158 Study. J Clin Oncol 2022; 40 (7): 752-761.

8 O'Malley DM, Bariani GM, Cassier PA et al. Health-related quality of life with pembrolizumab monotherapy in patients with previously treated advanced microsatellite instability high/mismatch repair deficient endometrial cancer in the KEYNOTE-158 study. Gynecol Oncol 2022; 166 (2): 245-253.

9 Makker V, Colombo N, Casado Herraez A et al. Lenvatinib plus Pembrolizumab for Advanced Endometrial Cancer. N Engl J Med 2022.

10 Cherny NI, Dafni U, Bogaerts J et al. ESMO-Magnitude of Clinical Benefit Scale version 1.1. Ann Oncol 2017; 28 (10): 2340-2366.
